# Complexes of Formaldehyde and α-Dicarbonyls with Hydroxylamine: FTIR Matrix Isolation and Theoretical Study

**DOI:** 10.3390/molecules26041144

**Published:** 2021-02-20

**Authors:** Barbara Golec, Magdalena Sałdyka, Zofia Mielke

**Affiliations:** 1 Institute of Physical Chemistry, Polish Academy of Sciences, Kasprzaka 44/52, 01-224 Warsaw, Poland; 2 Faculty of Chemistry, University of Wrocław, F. Joliot-Curie 14, 50-383 Wrocław, Poland; magdalena.saldyka@chem.uni.wroc.pl (M.S.); zofia.mielke@chem.uni.wroc.pl (Z.M.)

**Keywords:** carbonyls, hydroxylamine, hydrogen bond, matrix isolation, vibrational spectroscopy, computational chemistry

## Abstract

The interactions of formaldehyde (FA), glyoxal (Gly) and methylglyoxal (MGly) with hydroxylamine (HA) isolated in solid argon and nitrogen were studied using FTIR spectroscopy and ab initio methods. The spectra analysis indicates the formation of two types of hydrogen-bonded complexes between carbonyl and hydroxylamine in the studied matrices. The cyclic planar complexes are stabilized by O–H⋯O(C), and C–H⋯N interactions and the nonplanar complexes are stabilized by O–H⋯O(C) bond. Formaldehyde was found to form with hydroxylamine, the cyclic planar complex and methylglyoxal, the nonplanar one in both argon and nitrogen matrices. In turn, glyoxal forms with hydroxylamine the most stable nonplanar complex in solid argon, whereas in solid nitrogen, both types of the complex are formed.

## 1. Introduction

Chemical and photochemical reactions often proceed through many intermediate stages between the substrates and products. The reactive intermediates formed at the first stages are usually not observed at normal conditions but can be stabilized in an inert environment. The excellent technique for such a purpose is a low-temperature matrix isolation method, which enables the isolation of unstable intermediates in the rare gas matrix [1]. Many reactions begin with the formation of the molecular complexes between the substrates, which also can be isolated in the matrices. Using the FTIR spectroscopy and ab initio methods, we are able to distinguish the nature of interactions and the structures of the isolated complexes [2,3].

In this work, we used the FTIR matrix isolation spectroscopy and MP2 calculations to investigate the structures of the complexes formed between simple carbonyl (formaldehyde) and α-dicarbonyl compounds glyoxal and methylglyoxal, with hydroxylamine in argon and nitrogen environment. Identification and characterization of these complexes is the first step of our study on isolation of highly unstable intermediate, called hemiaminal, that is formed in oxime formation reaction [4,5] and will be the subject of our next paper. Oximes are widely used compounds in synthetic chemistry in biomedical fields, for example, in the coupling reactions of peptides, proteins, oligosaccharides, and oligonucleotides and in bioconjugation reaction [6,7,8,9,10,11]. On the other hand, to explain the acid-catalyzed reaction of nucleophiles with the carbonyl group, the formation of weak hydrogen bonds between the carbonyl group of aldehydes and ketones with proton donors has been postulated [12]. Therefore, the deep understanding of the hydrogen bond interaction of carbonyl compounds with nucleophilic hydroxylamine is an important issue.

Some binary complexes between formaldehyde or α-dicarbonyls and proton donors have been investigated. The most studied are the complexes of formaldehyde with water [13,14,15,16,17,18,19,20,21,22,23,24,25,26,27,28,29,30,31]. Additionally, there are some words about the interaction of formaldehyde with hydrogen halides and cyanide [32,33,34,35,36], formamide [37,38], nitroxyl [39], ammonia [33], methane [40], and methanol [41]. For α-dicarbonyls, the interactions of glyoxal, methylglyoxal and diacetyl with water [42], methanol [43,44], hydrogen peroxide [45,46], and hydroperoxyl radical [47] have been studied. The results of these investigations show that the water complexes of formaldehyde or α-dicarbonyls trapped in argon matrices are stabilized by the OH⋯O(C) hydrogen bond formed between water and carbonyl oxygen. The most stable structure of the methanol complex with formaldehyde is also predicted to be stabilized by a hydrogen bond. However, for the methanol complexes with α-dicarbonyls, the ab initio calculations predict the non-hydrogen-bonded structures as the most stable ones. Experimental data show that in the Ar matrix, glyoxal forms the non-hydrogen-bonded structure with methanol, but methylglyoxal and diacetyl form the planar cyclic structures stabilized by O–H⋯O(C) and C–H⋯O(C) interactions.

Hydroxylamine dimers and their complexes with water and ammonia isolated in argon and nitrogen matrices have been studied by Yao and Ford [48,49,50]. The dimer (NH_2_OH)_2_ has a cyclic structure stabilized by two O–H⋯N hydrogen bonds. In hydroxylamine complexes with H_2_O and NH_3,_ the OH group of NH_2_OH acts as a proton donor to the oxygen or nitrogen atom of water or ammonia, respectively. These complexes are relatively strong; the ν(OH) band of hydroxylamine shifts by 100 and 300 cm^−1^ to lower wavenumbers in the spectra of the H_2_O and NH_3_ complexes, respectively. A much weaker complex formed between hydroxylamine and carbon monoxide stabilized by O–H⋯C=O interaction has been observed in the Ar matrix. In this complex, the ν(OH) band shifts 39 cm^−1^ towards lower energies [51]. For the isocyanic acid-hydroxylamine complex, only the hydrogen-bonded structure with the NH group of HNCO attached to the oxygen atom of the NH_2_OH molecule was identified in solid argon [52].

## 2. Results and Discussion

### 2.1. Formaldehyde–hydroxylamine Complexes

**Figure 1:**
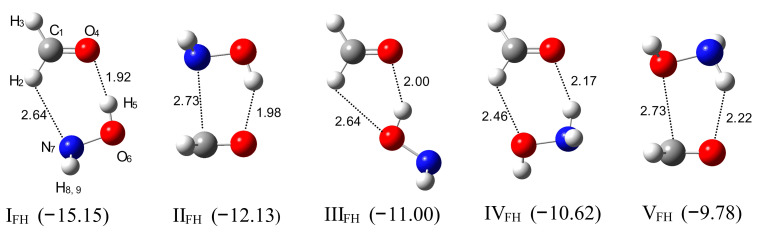
The optimized structures of the HCHO–NH_2_OH complexes, I_FH_–V_FH_. The ∆E_CP_(ZPE)-binding energies in kJ mol^−1^ are given in parentheses. The intermolecular distances are given in Å.

Ab Initio Calculations. The MP2/6-311++G(2d,2p) calculations predict the stability of five different structures of a hydrogen-bonded complex between formaldehyde and hydroxylamine with stoichiometry 1:1. Their structures and binding energies (ΔE^CP^(ZPE) in kJ mol^−1^) are presented in Figure 1 and Figure S1 in Supplementary Material.

The most stable complex, I_FH_, has a cyclic structure in which two subunits are stabilized by two hydrogen bonds: the relatively strong OH⋯O(C) bond (R(H⋯O) = 1.92 Å) and the weak CH⋯N interaction (R(H⋯N) = 2.64 Å). The selected structural parameters of this complex are listed in Table S1 in Supplementary Material. In this complex, the OH group of hydroxylamine acts as a proton donor and the nitrogen atom as a proton acceptor. The two hydrogen bonds are positioned in one plane, which is reflected by the value of the torsion angles φC1-O4-H5-O6 and φC1-H2-N7-O6 equal to 0 degrees. This structure is about 3 kJ mol^−1^ more stable than II_FH,_ which is stabilized by one hydrogen bond, OH⋯O(C), formed between the OH group of hydroxylamine and the oxygen atom of formaldehyde (R(H⋯O) = 1.98 Å). The next three structures, III_FH_, IV_FH_ and V_FH,_ have comparable energies (−11.00, −10.62, and −9.78 kJ mol^−1^, respectively). In III_FH,_ the hydroxylamine hydroxyl group interacts with formaldehyde forming OH⋯O(C) bond (R⋯O(C) = 2.0 Å) weaker than in I_FH_ and II_FH_. The structures IV_FH_ and V_FH_ are stabilized by NH⋯O(C) interaction between the NH group of hydroxylamine and an oxygen atom of formaldehyde.

**Figure 2:**
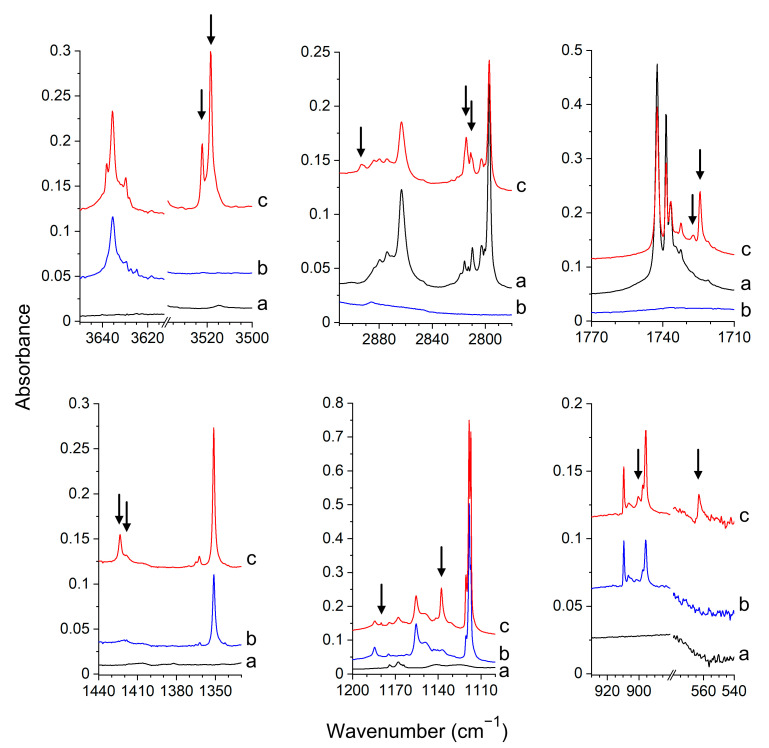
The spectra of the HCHO/Ar (**a**), NH_2_OH/Ar (**b**) and HCHO/NH_2_OH/Ar (**c**) matrices recorded after matrix deposition at 11 K. The bands of the HCHO–NH_2_OH complex are marked by the arrows.

Experimental Spectra. The infrared spectra of the formaldehyde and hydroxylamine molecules isolated in argon and nitrogen matrices agree with those previously reported [25,48,49,53,54,55,56]. When both FA and HA are trapped in the matrices, a set of new absorptions appears in the spectra that are not observed in the spectra of parent molecules. The selected regions of experimental spectra of FA/HA/Ar matrix are shown in Figure 2.

The new absorptions, indicated in the spectra by the arrows, are assigned to the HCHO–NH_2_OH complex. The splitting of the bands is assigned to the matrix cage effect as the observed spectral pattern of the complex differs in the spectra of argon and nitrogen matrices. In Table 1, the observed experimental wavenumbers and wavenumbers shifts are compared with the MP2 calculated values predicted for the two most stable forms of the formaldehyde–hydroxylamine complexes (I_FH_ and II_FH_). The large red-shift of the ν(OH) band of HA (−115.1 and −124.6 cm^−1^ in Ar and N_2_ matrices, respectively) and the red-shift of the ν(C=O) band of FA after complex formation (−16.8 and −17.1 cm^−1^) in Ar and N_2_ matrices, respectively indicate that the complex has the hydrogen-bonded structure with the NH_2_OH molecule attached to the carbonyl group of HCHO. The comparison of the experimental and theoretical spectra clearly shows that in both argon and nitrogen matrices, one type of complex is formed. The identified complex bands for the OH, C=O groups evidence that the I_FH_ structure is created in both matrices. The calculations indicate that for the structure I_FH,_ the strongest band corresponds to the ν(OH) stretching vibration (408 km mol^−1^). It is ca 3.5 times more intense than the other band of the OH group, namely τ(OH) (115 km mol^−1^). For the II_FH_ complex, both ν(OH) and τ(OH) bands are predicted to have comparable intensities (117, 122 km mol^−1^). Moreover, τ(OH) is distinctly more perturbed in I_FH_ than in II_FH_ (+238, +106 cm^−1^, respectively). In experimental spectra, the ν(OH) band is much more intense than the τ(OH) one, which indicates that the I_FH_ structure is formed. The observed shift of the τ(OH) vibration of HA (162.1 cm^−1^, Ar; 172.5 cm^−1^, N_2_) also corresponds better with the value +238 cm^−1^ predicted for the structure I_FH_ than with the value +106 cm^−1^ calculated for the complex II_FH_. The other calculated wavenumber shifts and intensities for the I_FH_ structure also match well the observed ones, as one can see in Table 1.

Nelander [25] studied the HCHO–H_2_O complex and found that the hydrogen-bonded structures stabilized by the O–H···O(C) bond are formed both in argon and nitrogen matrices. The wavenumbers shifts of the H_2_O vibrations for the complex isolated in solid argon were found to be −25.0 cm^−1^ for the ν_as_(OH) and −52.9, −57.6 cm^−1^ for the ν_s_(OH) vibrations, whereas ν(C=O) of FA was shifted by −5.2 cm^−1^ after complex formation. Such shifts pattern indicates that the HCHO–H_2_O complex is weaker than the HCHO–NH_2_OH one.

### 2.2. Glyoxal–hydroxylamine Complexes

**Figure 3:**
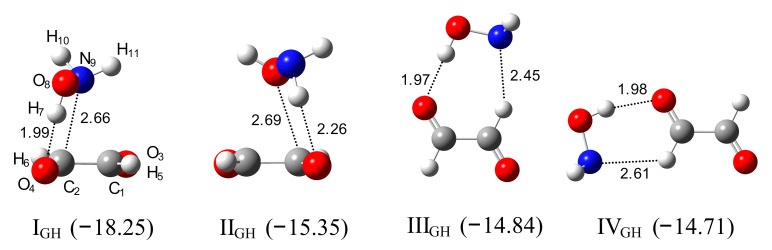
The optimized structures of the CHOCHO–NH_2_OH complexes, I_GH_–IV_GH_. The ∆E^CP^ (ZPE)-binding energies in kJ mol^−1^ are given in parentheses. The intermolecular distances are given in Å.

*Ab Initio Calculations*. Glyoxal in the standard conditions exists in a *trans* form [57,58,59], so exclusively, this conformer is considered in our study. The MP2 calculations show the stability of eight different complexes of 1:1 stoichiometry, which can be formed between Gly and HA. The four most stable ones are presented in Figure 3, and all complexes are shown in Figure S2 in Supplementary Material. The selected structural parameters of these complexes are listed in Table S2. The two most stable structures, I_GH_ and II_GH_, are the nonplanar hydrogen-bonded complexes in which the hydroxylamine moiety is placed above the plane of one of the carbonyl groups of glyoxal moiety. The I_GH_ complex with the binding energy of −18.25 kJ mol^−1^ is stabilized by the O–H⋯O(C) bond, as evidenced by the elongation of the O–H and C=O bonds (see Table S2). The intermolecular distance between the oxygen atom of glyoxal and the hydrogen atom of hydroxylamine has a length of 1.988 Å, and the O–H⋯O angle is equal to 148.2°. In turn, the II_GH_ complex (ΔE = −15.35 kJ mol^−1^) is stabilized by N-–H⋯O(C) interaction, which leads to elongation of the N–H and C=O bonds (see Table S2). The ab initio studies performed earlier for the glyoxal–methanol [43] and methylglyoxal–methanol complexes [44] indicated that the nonplanar non-hydrogen-bonded structures are the most stable ones. Decomposition of the interaction energy performed for the glyoxal–methanol complex [43] showed that the relative stability of the isomeric glyoxal complexes results from the subtle interplay between all energy components. However, the calculations indicated also that the dispersion energy contributed more to the stabilization of the non-hydrogen-bonded complex than to the hydrogen-bonded one.

The next six complexes are stabilized by two hydrogen bonds and have planar, cyclic structures. Four of them are sustained by the O–H⋯O(C) bond and additionally by C–H⋯N (III_GH_, IV_GH_) or C–H⋯O(N) interaction (V_GH_). Two complexes are stabilized by the N–H⋯O(C) and C–H⋯O(N) bonds (VI_GH_, VII_GH_). In III_GH_ and IV_GH_ complexes, the intermolecular distances between the oxygen atom of Gly and hydrogen atom of HA are equal to 1.970 Å, and 1.980 Å and the O–H⋯O angle values are equal to 176.9° and 163.3°, respectively. The CH⋯N bond lengths are predicted as 2.458 Å and 2.609 Å and angles as 151.5° and 118.9°.

**Figure 4:**
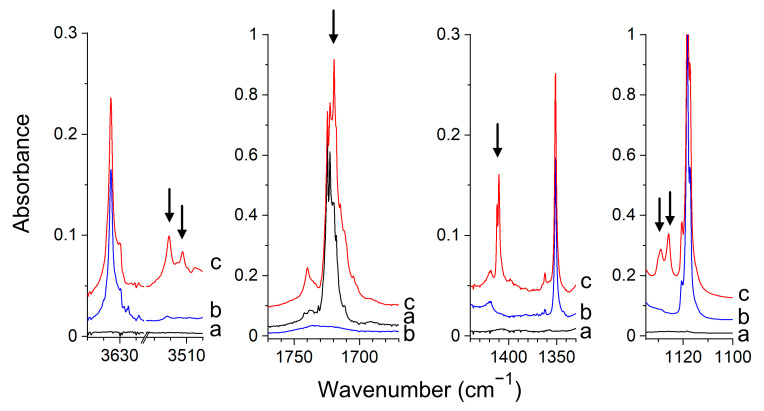
The spectra of the CHOCHO/Ar (**a**), NH_2_OH/Ar (**b**) and CHOCHO/NH_2_OH/Ar (**c**) matrices recorded after matrix deposition at 11 K. The bands of the CHOCHO–NH_2_OH complex are indicated by the arrows.

**Figure 5:**
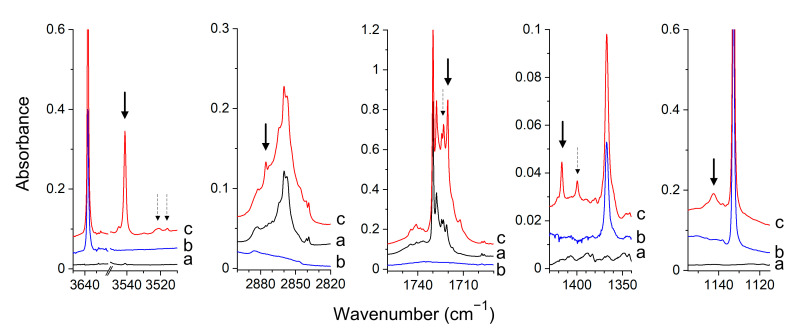
The spectra of the CHOCHO/N_2_ (**a**), NH_2_OH/N_2_ (**b**) and CHOCHO/NH_2_OH/N_2_ (**c**) matrices recorded after matrix deposition at 11 K. The bands of CHOCHO–NH_2_OH complexes are indicated by the arrows. Solid and dashed arrows correspond to the III_GH_ and I_GH_ structures, respectively.

*Experimental Spectra.* The infrared spectra of the Gly/Ar(N_2_), HA/Ar(N_2_) and d-HA/Ar(N_2_) matrices agree well with those reported in the literature [48,49,56,59,60]. In the spectra of the matrices doped both with glyoxal and hydroxylamine, a set of new absorptions appeared in the vicinity of Gly and HA or d-HA absorptions that can be assigned to the complexes formed between glyoxal and hydroxylamine. The selected regions of the spectra of Gly/HA/Ar and Gly/HA/N_2_ are presented in Figure 4 and Figure 5, respectively. The bands assigned to the glyoxal–hydroxylamine complexes are marked by the arrows. The wavenumbers of all observed complex bands are collected in Table 2 and Table S3 in Supplementary Material. The results obtained in experiments with the deuterated hydroxylamine are presented in Figures S3 and S4 and in Table S4 in Supplementary Material.

Based on the experimental data and calculated wavenumber shifts, we have assigned the new bands in the spectra of an argon matrix to the nonplanar I_GH_ structure. In the nitrogen matrix, both the doubly hydrogen-bonded cyclic structure corresponding to III_GH_ and the nonplanar I_GH_ complex were found formed, as discussed below. In the spectra of argon matrices, the ν(OH) band of the complexed hydroxylamine is distinctly shifted to lower wavenumbers, which suggests the formation of the hydrogen-bonded complex. The observed Δν(OH) value, −118.8 cm^−1^_,_ matches quite well the predicted value of −145 cm^−1^ for the structure I_GH_. The intensity of the ν(OH) band is comparable with the intensities of the other strong bands of the complex as predicted for I_GH_ by calculations. The observed, large shift (+60.9 cm^−1^) of the δ(NOH) band of hydroxylamine after complex formation agrees well with the calculated value for I_GH_, equal to +61.0 cm^−1^. In an experiment with deuterated hydroxylamine, the wavenumbers shifts for the ν(OD) and δ(NOD) modes are equal to −84.1 cm^−1^ and +33.5 cm^−1^, respectively. These values agree with the calculated ones equal to −106 cm^−1^ and +38 cm^−1^. The other experimental wavenumber shifts of the identified complex bands also match well those calculated for the I_GH_ structure.

The noticeable difference between the spectra of the complex trapped in solid argon and the one trapped in solid nitrogen is strong ν(OH) absorption in nitrogen spectra, which is much more intense than the other bands of the complex (see Figure 5). This is not the case for the argon spectra. For example, the estimated experimental intensity ratios (I_νOH_/I_δNOH_)_exp_, (I_νOH_/I_ωNH2_)_exp_ are equal to ca. 0.8, 0.4 for the complex identified in the argon matrix, and they increase to ca. 12, 4, respectively, for the complex trapped in the nitrogen matrix. Such experimental intensity ratios match well the calculated ones for the ν(OH) absorptions of the nonplanar I_GH_ and cyclic planar III_GH_ (IV_GH_) complexes. The calculated ratios (I_νOH_/I_δNOH_)_calc_, (I_νOH_/I_ωNH2_)_calc_ are equal to ca. 1.2, 0.7 for I_GH_ and ca. 13, 3.5 for III_GH_ (for IV_GH_ similar intensity ratios are obtained as for III_GH_). The observed ν(OH) shift for the complex trapped in nitrogen (−96.5 cm^−1^) is slightly less than that for the complex in argon (−118.8 cm^−1^), which is in accord with the shifts predicted for III_GH_, IV_GH_ and I_GH_ (−106, −103, −145 cm^−1^_,_ respectively). The experimental shift of the ν(CH) vibration (+18.5 cm^−1^) suggests that the III_GH_ complex and not the IV_GH_ one is created in the nitrogen matrix. The +18.5 cm^−1^ shift matches better with the +15 cm^−1^ value predicted for the III_GH_ structure than with the +1 value calculated for IV_GH_. The wavenumber shifts of the other identified vibrations are also in accord with the calculated ones for the III_GH_ structure (see Table 2 and Table S3). The significant red-shift of ν(OH) of glyoxal accompanied by a noticeable blue shift of ν(CH) after complex formation indicates that the III_GH_ complex isolated in solid nitrogen is stabilized both by the O–H···O(C) hydrogen bond and by blue-shifting C–H···N hydrogen bond. The blue shift of the ν(X-H) stretching wavenumber of the proton donor after complex formation is characteristic of the blue-shifting hydrogen bonding, also called the improper hydrogen bond. These bonds, formed mainly by the CH proton donors, have been a subject of intense theoretical [61,62,63,64,65,66] and experimental studies [65,66].

All the bands identified for the I_GH_ complex in the spectra of the argon matrices, as well as all the bands assigned to the III_GH_ complex in the spectra of solid nitrogen, exhibited the same intensity ratios in all performed experiments as expected for the bands due to the same species. However, in the spectra of the CHOCHO/NH_2_OH/N_2_ matrices, in addition to the bands attributed to III_GH_ (marked by solid arrows in Figure 5), three additional bands appeared (marked by dashed arrows in Figure 5) whose relative intensities were the same with respect to each other in the performed experiments however differed with respect to the absorptions assigned to III_GH_. The bands appeared as a very weak doublet at 3520.9, 3515.8 cm^−1,^ and as single absorptions at 1723.1, 1399.7 cm^−1^ in the region of the ν(OH), ν(C=O) and δ(NOH) vibrations, respectively. The shifts of these bands with respect to the corresponding absorptions of the parent molecules (Δν(OH)= −116.7, −121.8 cm^−1^, Δν(C=O) = −7.0 cm^−1^ and δ(NOH) = +32.4 cm^−1^) and their relative intensities indicate that they are due to the CHOCHO–NH_2_OH complex of the I_GH_ structure. The concentration ratio of the complexes III_GH_/I_GH_ was estimated on the basis of the ν(OH) and δ(NOH) absorptions ((I_exp_(III_GH_)/I_exp_(I_GH_)) × (I_calc_(I_GH_)/I_calc_(III_GH_)) and was found to be ca. 3:2. The change of the conditions of matrix deposition (concentration, depositions temperature) slightly affected the mutual concentration of the two structures.

Mucha and Mielke studied the glyoxal complexes with water and hydrogen peroxide in the argon matrices [42,45]. Gly–H_2_O and Gly–H_2_O_2_ complexes isolated in solid argon have planar, cyclic structures analogous to the III_GH_ one. The observed shifts of the ν(C–H), ν(C=O) wavenumbers of glyoxal are equal to +2.5, −0.6 cm^−1^, respectively, for the Gly–H_2_O complex; and to +28.9, +3.3 cm^−1^ (ν_s_(CH), ν_as_(CH)), −5.0 cm^−1^ (ν(C=O)) for the Gly–H_2_O_2_ one as compared to +18.5, −9.9 cm^−1^, respectively, for Gly-NH_2_OH, III_GH_. The observed perturbations of glyoxal vibrations in the above complexes indicate that the glyoxal complex with hydroxylamine is much stronger than that with water, and its strength is comparable to the complex with hydrogen peroxide. This is in accord with the calculated energy values for the above complexes (ca. −11, −17 and −18 kJ mol^−1^ for Gly–H_2_O, Gly−NH_2_OH and Gly–H_2_O_2_, respectively) [42,45].

### 2.3. Methylglyoxal–Hydroxylamine Complexes

**Figure 6:**
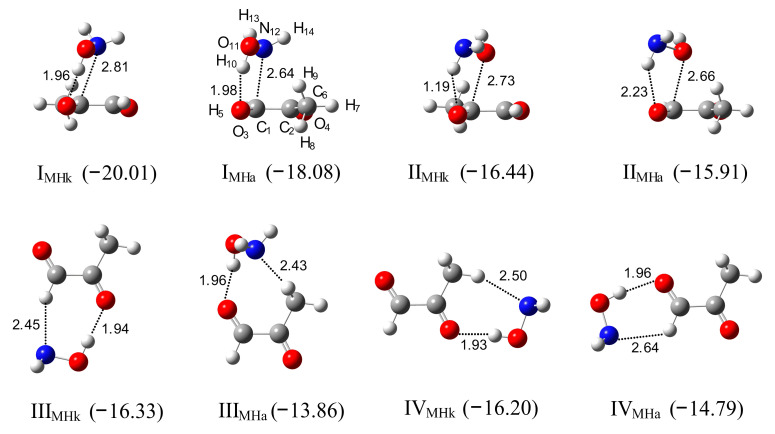
The optimized structures of the CH_3_COCHO–NH_2_OH complexes, I_MHk_–IV_MHk_ and I_MHa_–IV_MHa_. The intermolecular distances are given in Å. The ∆E^CP^(ZPE)-binding energies in kJ mol^−1^ are given in parentheses.

*Ab Initio Calculations*. Methylglyoxal, like glyoxal, in the standard conditions exists only as of the *trans* isomer [67], so only this conformer is considered in our study. The predicted structures of the complexes formed between methylglyoxal and hydroxylamine are presented in Figure 6 (eight most stable structures) and in Figure S5 in Supplementary Material (all fifteen structures). The geometrical parameters are listed in Table S5.

The structures of the MGly–HA complexes are analogous to the structures predicted for the Gly–HA ones. The two most stable structures, I_MHk_ and I_MHa_, with the binding energies of about −20 and −18 kJ mol^−1^, are nonplanar. In I_MHk,_ the hydroxylamine moiety is placed above the acetyl group and in I_MHa_ above the aldehyde group of methylglyoxal. These complexes are stabilized by the O–H⋯O(C) interaction between the oxygen atom of the aldehyde or acetyl group of methylglyoxal and the OH group of hydroxylamine. The formation of the hydrogen bond is manifested by the elongation of the O–H bond of hydroxylamine from 0.959 Å to 0.968 Å. The intermolecular H⋯O distances and O–H⋯O(C) angles are equal to 1.956 Å, 151.5° for I_MHk_ and 1.980 Å, 148.6° for I_MHa_. The nonplanar structures II_MHk_ and II_MHa_ are stabilized by N–H⋯O(C) interaction. The other predicted complexes have cyclic forms stabilized by two hydrogen bonds. Structures III_MHk_, III_MHa_, IV_MHk_, IV_MHa_, V_MHk_, V_MHa_, VI_MHk_ and VI_MHa_ are stabilized by the O–H⋯O(C) and, additionally, by (O)C–H⋯N or H_2_C–H⋯N interactions and the complexes VII_MHk_, VIII_MHk_, VIII_MHa_ by the N–H⋯O(C) hydrogen bond and by (O)C–H⋯O or H_2_C–H⋯N interaction.

**Figure 7:**
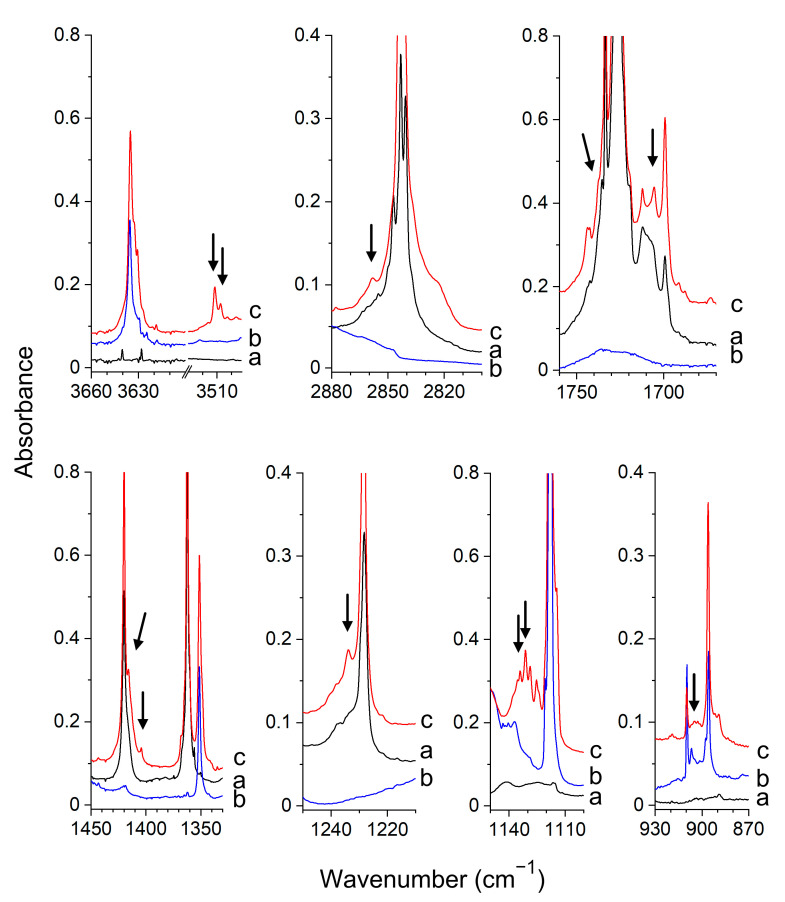
The spectra of the CH_3_COCHO/Ar (**a**), NH_2_OH/Ar (**b**) and CH_3_COCHO/NH_2_OH/Ar (**c**) matrices recorded after matrix deposition at 11 K. The bands of the CH_3_COCHO–NH_2_OH complex are indicated by the arrows.

**Figure 8:**
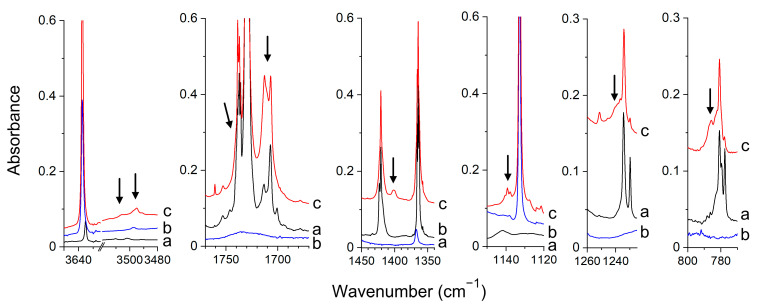
The spectra of the CH_3_COCHO/N_2_ (**a**), NH_2_OH/N_2_ (**b**) and CH_3_COCHO/NH_2_OH/N_2_ (**c**) matrices recorded after matrix deposition at 11 K. The bands of CH_3_COCHO–NH_2_OH complex are indicated by the arrows.

*Experimental Spectra.* In Figure 7 and Figure S6 (Supplementary Material), the infrared spectra of doubly doped MGly/HA/Ar, MGly/d-HA/Ar matrices are compared with the singly doped MGly/Ar, HA/Ar, d-HA/Ar matrices. In Figure 8, the result of the experiment carried in solid nitrogen is presented. The infrared spectra of MGly agree well with those reported earlier [42,60,68]. The new bands that appeared in the spectra of doubly doped matrices are marked by the arrows and are assigned to the MGly–HA, I_MHa_ complex.

Analysis of the spectra shows that in doubly doped argon and nitrogen matrices, the same type of hydrogen-bonded complex is formed. The comparison of our experimental data with the theoretical ones (see Table 3, Tables S6 and S7 in Supplementary Material) indicates that the isolated complex has a structure I_MHa_, as discussed below. The new band due to the ν(OH) vibration of the MGly–HA complex is shifted 125.8, 137.1 cm^−1^ towards lower wavenumbers in the spectra of argon and nitrogen matrices, respectively. The red-shift of the ν(OH) is accompanied by the blue shift of δ(NOH). The latter vibration is coupled in the complex with the perturbed δ(CH_3_) vibration. The coupling is supported by the CH_3_COHCO–ND_2_OD spectra in which neither of the two bands is observed (when δ(NOD) is shifted to lower wavenumbers and not coupled with δ(CH_3_), the latter band may be too weak to be observed). Two bands that are assigned to the coupled δ(NOH) + δ(CH_3_) vibrations appear at 1404.1, 1416.1 cm^−1^ in the spectra of an argon matrix. In the nitrogen matrix, only one new band was identified in the region of the δ(NOH), which is shifted +34.1 cm^−1^ from the corresponding monomer band. The above facts indicate that the OH group of hydroxylamine interacts with the methylglyoxal molecule forming the O–H⋯O(C) hydrogen bond and allow to exclude all structures in which this bond is not formed (II_MHa_, II_MHk_, VII_MHk_, VIII_MHa_, VIII_MHk_). The intensity of the ν(OH) band is comparable (or slightly larger) to the intensities of the other most intense bands (δ(NOH), ω(NH_2_), ν(C=O)) of MGly–HA complex in the spectra of Ar and N_2_ matrices as can be seen in Figure 7 and Figure 8. This is in accord with the predicted intensities for the I_MHa_, I_MHk_ complexes and allows us to eliminate the cyclic structures III_MHa_, III_MHk_, IV_MHa_, IV_MHk_ for, which the ν(OH) is predicted to be ca. 3.5–5 times more intense than the second most intense band of the complex (see Table 3 and Table S6). The other spectral features of the recorded spectra point to the presence of I_MHa_ in the matrix. The observed shifts of the ν_ket_(C=O) and ν_ald_(C=O) bands of MGly after complexation are equal to +4.2 and −21.0 in Ar (+3.9 and −18.2 cm^−1^ in N_2_ matrix, respectively), which suggests that in the complex the OH group of HA is interacting with the oxygen atom of the aldehyde group and not with acetyl one. The corresponding calculated shifts of the ν_ket_(C=O) and ν_ald_(C=O) bands are equal to +4, −10 cm^−1^ for I_MHa_ and −5, +6 cm^−1^ for I_MHk_, respectively. The observed shift of the CH stretch of the aldehyde group (+16.3 cm^−1^ in Ar) confirms that I_MHa_ is formed and not I_MHk_. The calculated Δν shifts for CH stretch vibration are equal to −1, + 24 cm^−1^ for I_MHk_, I_MHa_, respectively. The observed shifts of all other bands identified for the complex present in the Ar, N_2_ matrices also match well the predicted ones for the I_MHa_ complex.

We have found no sign of formation of any cyclic MGly–HA complex. In contrast, the interaction of methylglyoxal with water and methanol forms exclusively the cyclic planar complexes stabilized by the O–H⋯O(C) and C–H⋯O hydrogen bonds between the OH group of H_2_O or CH_3_OH and the acetyl or aldehyde oxygen atom of CH_3_COCHO [42,44]. The presence of the less stable complex I_MHa_ than the more stable I_MHk_ is probably due to the steric effects between the hydroxylamine moiety and methyl group of methylglyoxal.

### 2.4. AIM Analysis

In Figures S7 and S8 (Supporting Information), the location of the bond critical points (BCP) and ring critical points (RCP) in all optimized structures of the formaldehyde–hydroxylamine and glyoxal–hydroxylamine complexes are presented. The AIM parameters of intermolecular BCPs are collected in Table 4. The general classification of the interaction type can be performed using topological parameters [69,70]. The investigation of Laplacian of electron density (∇^2^ρ_b_) indicates if there is a local concentration (∇^2^ρ_b_ < 0) or a local depletion (∇^2^ρ_b_ > 0) of charge. Low ρ_b_ and ∇^2^ρ_b_ values and total electron energy H ≈ 0 indicate that the complexes are stabilized by weak hydrogen bonds or van der Waals interaction. The criteria for the presence of hydrogen bond limit the lower values of the electron density, ρ_b_, and the Laplacian of the electron density, ∇^2^ρ_b_, to 0.002, 0.024 a.u., respectively, and the corresponding upper values to 0.034, 0.139 a.u.

The inspection of the results presented in Figure S7 and Table 4 shows that the HCHO–NH_2_OH complexes are stabilized by OH⋯O, NH⋯O, CH⋯O and CH⋯N interactions. In two complexes, the N⋯C or O⋯C interaction occurs (II_FH_, V_FH_, respectively). For the OH⋯O and NH⋯O interactions, the ρ_b_ values are in the range 0.0136 < ρ_b_ < 0.0256 a.u, and positive Laplacian ∇^2^ρ_b_, is in the range 0.0513 < ∇^2^ρ_b_ < 0.0855 a.u., such values are characteristic of hydrogen bonds. The structures I_FH_, II_FH_, IV_FH_ and V_FH,_ are characterized by two intermolecular bond critical points, BCP, and one ring critical point, RCP. The structure III_FH_ involves only one BCP on the bond path corresponding to the interaction between the oxygen atom of formaldehyde and the hydrogen atom of the hydroxyl group of the hydroxylamine molecule. For the bonding in which the hydrogen atom of the CH group is involved (structures I_FH_ and IV_FH_), the electron densities values fall within the range: 0.009 < ρ_b_ < 0.01 a.u., and the Laplacian in the range: 0.0268 < ∇^2^ρ_b_ < 0.0337 a.u. These parameters indicate that a very weak hydrogen bond is responsible for the formation of CH⋯N and CH⋯O interactions. Data in Table 4 show that the highest values of the discussed parameters occurred for the complex I_FH,_ which is the most stable one according to calculations.

The topological analysis indicates that the structures of the CHOCHO–NH_2_OH complex are maintained by similar types of interactions as the configurations of the hydroxylamine complex with formaldehyde. In the glyoxal complexes, like in the formaldehyde ones, one BCP point corresponds to the OH···O or NH···O interaction and the second one to the CH···N or CH···O interaction which involves the hydrogen atom one of the two CH groups. In the nonplanar glyoxal complexes, one BCP corresponds to the OH···O or NH···O hydrogen bonding and the second one to the N···C or O···C interaction (I_GH_, II_GH_). The AIM parameters of BCPs along OH···O in the glyoxal complexes (I_GH_, III_GH_, IV_GH_, V_GH_, VIII_GH_) have very close values: ρ_b_ ≈ 0.02 a.u., ∇^2^ρ ≈ 0.07 a.u. as well as the: ρ_b_ ≈ 0.013 a.u., ∇^2^ρ ≈ 0.05 a.u. values calculated for the NH···O bond paths (II_GH_, VI_GH_, VII_GH_). It is interesting to notice that the structures I and II of the CHOCHO–NH_2_OH complexes, like the structures II and V of the HCHO–NH_2_OH system, involve one BCP on the bond path corresponding to the interaction between the nitrogen or oxygen atoms of hydroxylamine and the carbon atom of the glyoxal or formaldehyde molecule. The BCPs parameters for the bond paths involving CH···O or CH···N interactions in the CHOCHO–NH_2_OH complex have slightly larger values (ρ_b_ ≈ 0.01 a.u. and ∇^2^ρ ≈ 0.04 a.u.) than those characterizing analogous interactions in the HCHO–NH_2_OH complex.

The classification of the OH···O, NH···O interactions in all configurations of the HCHO–NH_2_OH and CHOCHO–NH_2_OH complexes as a hydrogen bonding interaction is strongly confirmed both by experimental and theoretical data and is free from doubt. The electron density and Laplacian values in the BCPs on the O···H and N···H bond paths are within the range characteristic for the hydrogen bonding. The large perturbations of the OH or NH stretching vibrations accompanied by perturbations of the carbonyl group vibrations, as demonstrated in the recorded infrared spectra of the hydroxylamine complexes, provide strong evidence of the formation of the O–H···O or N–H···O bonds. The other important interactions stabilizing the studied complexes are the ones in which the hydrogen atoms of the CH group of the carbonyl molecule are involved.

### 2.5. Hydrogen Bonding in the Carbonyl-Hydroxylamine Complexes

The results of our experimental and theoretical studies show that all studied carbonyl compounds (formaldehyde, glyoxal and methylglyoxal) form the hydrogen-bonded complexes with hydroxylamine in the argon and nitrogen matrices. The structures attributed to the complexes are gathered in Figures S9 and S10 in Supporting Information. FA was found to form the cyclic planar structure, I_FH_, and MGly the nonplanar one, I_MHa_, in both argon and nitrogen matrices. In turn, Gly forms the nonplanar complex, I_GH_, in the argon matrix, whereas in the nitrogen matrix, both the cyclic planar, III_GH_, structure and the nonplanar one are created.

The topological analysis indicated the following types of hydrogen bonding in the hydroxylamine complexes with formaldehyde and glyoxal, namely: OH···O, NH···O and CH···O, CH···N. In addition, in two FA complexes and two Gly ones, the analysis indicated the N···C and O···C interactions (II_FH_, I_GH_ and V_FH_, II_GH_, respectively). The most stable HCHO–NH_2_OH complex, I_FH_, observed in the argon and nitrogen matrices, is stabilized by two hydrogen bonds, OH···O and CH···N. In turn, the most stable CHOCHO–NH_2_OH complex, I_GH_, is stabilized by the OH···O hydrogen bond and N···C interaction; this complex is formed both in argon and nitrogen matrix. In the nitrogen matrix, the less stable, cyclic complex, III_GH_, stabilized by two hydrogen bonds, OH···O and CH···N is also created. Comparison of the predicted structural parameters for the isolated complexes shows that in the cyclic planar complexes, I_FH_, III_GH_, the OH⋯O(C) intermolecular distance is slightly shorter (0.02–0.06 Å) than in the nonplanar structures, I_GH_, I_MHa_. The formation of the cyclic structures is accompanied by a strong increase of ν(OH) intensity in contrast with the nonplanar ones.

In Table 5, the wavenumber shifts of the hydroxylamine vibrations in the complexes with various proton acceptors are compared. It can be observed that in the complexes with formaldehyde, glyoxal and methylglyoxal, the perturbation of hydroxylamine vibrations is quite similar and shows small sensitivity to the carbonyl molecule and to the complex structure. The shifts for carbonyl complexes are comparable with the data reported for the NH_2_OH-H_2_O complex [49] and are much smaller (two/three times) than the values reported for the hydroxylamine complexes with NH_3_ [50] and for the hydroxylamine dimer (NH_2_OH)_2_ [48] in accord with much stronger interaction in the latter complexes. The smallest perturbation of hydroxylamine vibrations is observed for the NH_2_OH-CO complex identified in the argon matrix, which is much weaker than the complexes of hydroxylamine with formaldehyde and α-dicarbonyls [51].

Over the many past decades, there have been many attempts to decode from vibrational spectra the information on the electronic structure of a molecule and about the strength of its bonds. Such information can now be obtained from the local vibrational mode analysis that was originally introduced by Konkoli and Cremer and is becoming increasingly popular [71]. The method was successfully applied to various systems, among them to a number of hydrogen-bonded systems, mainly to homo-aggregates [71,72,73,74]. It would be very interesting to apply the local vibrational mode analysis to study the heterodimers between hydroxylamine α-dicarbonyls in order to get a deeper insight into the bonding of these systems, and such study is planned to be performed in the future.

## 3. Materials and Methods

Formaldehyde, HCHO (FA), was prepared by heating paraformaldehyde (95%, Sigma-Aldrich) up to 65–75 °C directly in the deposition line. Glyoxal, CHOCHO (Gly), was prepared by heating the solid trimer dihydrate (98%, Sigma-Aldrich: St. Louis, MO, USA) topped with phosphorus pentoxide (P_4_O_10_) powder under vacuum to 120 °C and collecting the gaseous monomer in a trap at 77 K. Methylglyoxal, CH_3_COCHO (MGly), was obtained from 40% aqueous solution of methylglyoxal (Sigma-Aldrich). The major amount of water was distilled off in a vacuum line; then, the sample was depolymerized by heating at about 90 °C. Next, the gaseous monomeric methylglyoxal was passed through P_4_O_10_ and trapped in 77 K. The sample was stored at liquid nitrogen temperature. Hydroxylamine, NH_2_OH (HA), was generated by heating (at 50–65 °C) the hydroxylamine phosphate salt (95%, Fluka: Buchs, Switzerland) directly in the deposition line. Deuterated hydroxylamine, ND_2_OD (d-HA), was prepared by heating hydroxylamine phosphate salt in D_2_O solution and evaporating water in a vacuum. This procedure was repeated several times until the deuteration degree was about 90%.

The carbonyl/hydroxylamine/argon (or nitrogen) matrices were prepared by simultaneous deposition of carbonyl/Ar(N_2_) and NH_2_OH vapor on a cold gold mirror held at 11–17 K by a closed-cycle helium refrigerator (Displex 202A, Air Products: Allentown, PA, USA). The carbonyl/Ar(N_2_) concentration was varied in a range of 1/100–1/2000. The absolute concentration of NH_2_OH in the matrices could not be determined, but its concentration was varied by changing the rare gas flow rate and the heating temperature of the hydroxylamine salt. The infrared spectra with a resolution of 0.5 cm^−1^ were recorded in a reflection mode with Bruker 113v FTIR spectrometer using a liquid-nitrogen-cooled MCT detector.

The bands that are assigned to the 1:1 complexes between carbonyl and hydroxylamine were observed already in the spectra of the diluted matrices; their relative intensities with respect to the other species trapped in the matrix decreased after matrix annealing and when the matrix concentration increased. The relative intensities of all bands assigned to one type of the 1:1 complex were constant in all performed experiments. Matrix annealing increased the concentration of the HA dimers and higher-order complexes and obscured the spectra.

The MP2 method with 6-311++G(2d,2p) basis set was used for geometry optimization of the structures and calculation of harmonic vibrational spectra of the monomers and hydroxylamine (HA- and d-HA-substituted) complexes with FA, Gly and MGly [75,76]. All the presented monomer and complex structures correspond to the real minima on PES as indicated by the positive values of their calculated wavenumbers. Binding energies were corrected by the Boys–Bernardi full counterpoise procedure [77]. The calculations were performed using the Gaussian 03 program [78]. The topological analysis of the electron density was performed with the AIMAll program [79].

## 4. Conclusions

The FTIR matrix isolation spectroscopy and MP2/6-311++G(2d,2p) calculations were applied to investigate the complexes of formaldehyde and α-dicarbonyls (glyoxal, methylglyoxal) with hydroxylamine in solid argon and nitrogen matrices. The results of the study show that NH_2_OH forms with carbonyls two types of hydrogen-bonded complexes. One type involves the nonplanar structures stabilized by the O–H⋯O(C) interactions in which the hydroxylamine moiety is placed above the carbonyl group of formaldehyde or above one of the two C=O groups of α-dicarbonyls. The ab initio calculations predict this structure as the most stable form of the 1:1 complex between α-dicarbonyl and hydroxylamine. The other type includes the planar cyclic structure stabilized by the O–H⋯O(C) and C–H⋯N hydrogen bonds. This structure is predicted to be the most stable one for HCHO–NH_2_OH and was observed for this complex isolated in both argon and nitrogen matrices. The structure of the glyoxal–hydroxylamine complex is affected by the matrix in which it is isolated. In the argon matrix, the most stable nonplanar complex is formed, whereas in solid nitrogen, both the cyclic planar structure, corresponding to one of the local minima and the most stable nonplanar structure, are created. In turn, the methylglyoxal–hydroxylamine complex forms the most stable nonplanar structure in both argon and nitrogen matrices.

The obtained data confirm that the matrix can influence the nature of the isolated complex and can stabilize the less stable structures as it was observed for the CHOCHO–NH_2_OH complex. The formation of the less stable complex in matrices is attributed to the dipole–dipole interactions that dominate at long-range geometries and direct the two approaching submolecules in the matrix to one of the energy minima. It is not excluded that the carbonyl complexes with hydroxylamine are formed in a similar way [80]. This kind of process was observed for the formation of formic acid dimers [81], acetohydroxamic acid [82] and N-hydroxyurea dimers [83] or *N,N*-dimethylformamide complexes with water and ammonia in solid argon [84].

## Figures and Tables

**Table 1:** The comparison of the observed wavenumbers (cm^−1^) and wavenumber shifts (Δν = νFH − νM) for the HCHO–NH_2_OH (FH) complexes present in the argon (Ar) and nitrogen (N_2_) matrices with the corresponding values calculated for the structures I_FH_ and II_FH_. In parentheses, the calculated band intensities are given (km mol^−1^).

Approximate Description	Experimental						Calculated	
	Ar			N_2_			Δν	
	ν M	νFH	Δν ^1^	ν M	νFH	Δν ^1^	I_FH_	II_FH_
**Hydroxylamine**								
**ν(OH)**	3635.5	3522.2 3518.5	−115.1	3637.6	3516.0 3510.0	−124.6	−141 (408)	−126 (117)
**δ(NOH)**	1351.2	1423.4 1418.7	+69.9	1367.4	1427.7	+60.3	+86 (36)	+62 (51)
**ω(NH_2_)**	1118.3	1137.8	+19.5	1133.0	1156.7	+23.7	+24 (117)	+13 (102)
**ν(NO)**	895.6	900.5	+4.9	895.3	897.0 901.8	+4.1	+6 (7)	+8 (4)
**τ(OH)**	402 ^2^	562.1	+162.1	402 ^2^	574.5	+172.5	+238 (115)	+106 (122)
**Formaldehyde**								
**ν_as_(CH)**	2864.1	2893.3 2891.1	+28.1	2866.4	2892.3	+25.9	+56 (34)	+34 (72)
**ν_s_(CH)**	2798.0	2814.2 2811.0	+14.6	2800.1 2798.0			+25 (77)	+29 (56)
**ν(C=O)**	1742.2	1727.1 1724.4	−16.8	1740.7 1739.7	1723.1	−17.1	−20 (53)	−9 (66)
**δ(CH_2_)**	1499.1	1493.9	−5.2	1499.6 1496.4			−6 (15)	−3 (14)
**γ(CH_2_)**	1168.6	1179.8	+11.2	1170.0 1167.9	1180.0	+11.0	+12 (5)	+8 (20)

^1^ In the case when the splitting of the band was observed, the average of the two wavenumbers at which the two peaks appear was taken into account to calculate Δν value. ^2^ Gas-phase data [56].

**Table 2:** The comparison of the observed wavenumbers (cm^−1^) and wavenumber shifts (Δν = νGH − νM) for the CHOCHO–NH_2_OH (GH) complexes present in the Ar and N_2_ matrices with the corresponding calculated values for the complexes I_GH_, III_GH_ and IV_GH_. In parentheses, the calculated band intensities are given (km mol^−1^).

Approximate Description	Experimental						Calculated		
	Ar			N_2_			Δν		
	ν M	νGH	Δν ^1^	ν M	νGH ^2^	Δν ^1^	I_GH_	III_GH_	IV_GH_
**Hydroxylamine**									
**ν(OH)**	3635.5	3521.0 3512.4	−118.8	3637.6	3541.1 *3520.9* *3515.8*	−96.5 *−119.2*	−145 (7)	−106 (383)	−103 (356)
**δ(NOH)**	1351.2	1412.1 1410.2	+60.9	1367.4	1416.6 *1399.7*	+49.2 *+32.3*	+61 (63)	+72 (29)	+71 (34)
**ω(NH_2_)**	1118.3	1129.0 1125.8	+9.1	1133.0	1142.6	+9.6	+14 (11)	+24 (112)	+22 (122)
**ν(NO)**	895.6			895.3	898.8	+3.5	+14 (3)	+9 (8)	+6 (8)
**Glyoxal**									
**ν(CH)**	2860.1 2854.9	2857.6	−0.4	2857.1	2875.6	+18.5	−9 (60) +12 (39)	+15 (53) +20 (1)	+1 (52) +51 (8)
**ν(C=O)**	1724.5	1719.0	−5.5	1730.1	1720.2 *1723.1*	−9.9 −7.0	−4 (122) +4 (23)	−11 (126) −6 (20)	−13 (89) −6 (33)
**γ(CH)**	812.1 807.8			807.4	820.8	+13.4	+37 (0)	+23 (8)	+16 (2)

^1^ In the case when the splitting of the band was observed, the average of the two wavenumbers at which the two peaks appeared was taken into account to calculate Δν value. ^2^ The wavenumbers in italic are due to complex of different structure (see text).

**Table 3:** The comparison of the observed wavenumbers (cm^−1^) and wavenumber shifts (Δν = νMH − νM) for the CH_3_COCHO–NH_2_OH (MH) complexes present in the Ar and N_2_ matrices with the corresponding calculated values for the complexes I_MHk_ and I_MHa_. In parentheses, the calculated band intensities are given (km mol^−1^).

Approximate Description	Experimental						Calculated	
	Ar			N_2_			Δν	
	ν M	ν MH	Δν ^1^	ν M	ν MH	Δν ^1^	I_MHk_	I_MHa_
**Hydroxylamine**								
**ν(OH)**	3635.5	3511.7 3507.6	−125.8	3637.6	3505.7 3495.2	−137.1	−148 (138)	−156 (73)
**δ(NOH) ^2^**	1351.2	1404.1	+52.9	1367.4	1402.7 1400.3	+34.1	+64 (58)	+62 (51)
**ω(NH_2_)**	1118.3	1134.1 1131.2	+14.4	1133.0	1139.3 1140.6	+7.0	+17 (117)	+11 (109)
**ν(NO)**	895.6	904.8 902.9	+8.3	895.3			+9 (5)	+13 (6)
**Methylglyoxal**								
**ν(CH)**	2843.1 2840.7	2858.2	+16.3	2844.7 2840.2 2835.9			−1 (60)	+24 (51)
**ν*_ket_*(C=O)**	1733.5	1737.7	+4.2	1739.0 1737.2 1735.9	1741.3	+3.9	−5 (132)	+4 (61)
**ν*_ald_*(C=O)**	1726.4	1705.4	−21.0	1730.1 1727.9	1710.8	−18.2	+6 (27)	−10 (97)
**δ(CH_3_) ^2^**	1420.0	1416.1	−3.9	1423.2 1420.8			0 (8) +3 (24) −3 (36)	+5 (6) +3 (25) −2 (31)
**ν_as_(C–C)**	1228.3	1233.7	+5.4	1234.5 1229.9	1240.5 1237.1	+6.6	+5 (14)	+5 (15)
**ν_s_(C–C)**	777.1	784.7	+7.6	780.9	785.8	+4.8	+6 (15)	+6 (15)
				779.5	782.3			
				777.6				

^1^ In the case when the splitting of the band was observed, the average of the two wavenumbers at, which the two peaks appear was taken into account to calculate Δν value. ^2^ The 1416.1, 1404.1 cm^−1^ bands observed in the spectra of the complex in Ar matrix are assigned to the coupled δ(NOH)+δ(CH_3_) vibrations.

**Table 4:** AIM properties of selected critical points computed at the MP2/6-311++G(2d,2p) level. All data reported in atomic units.

Complex	BCP		ρ_b_		∇^2^ρ_b_		Complex		BCP	ρ_b_	∇^2^ρ_b_
Formaldehyde–Hydroxylamine Complexes							Glyoxal–Hydroxylamine Complexes				
I_FH_		O4-H5 ^a^		0.0256		0.0855	I_GH_	C2-N9		0.0203	0.0626
		H2-N7 ^c^		0.0090		0.0268		O4-H7 ^a^		0.0213	0.0800
II_FH_		O4-H5 ^a^		0.0207		0.0791	II_GH_	C1-O8		0.0158	0.0582
		C1-N7		0.0160		0.0514		O3-H10 ^b^		0.0129	0.0472
III_FH_		O4-H5 ^a^		0.0226		0.0798	III_GH_	O4-H7 ^a^		0.0225	0.0772
								H5-N9 ^d^		0.0122	0.0342
IV_FH_		O4-H8 ^b^		0.0164		0.0560	IV_GH_	O4-H7 ^a^		0.0227	0.0782
		H2-O6 ^c^		0.0099		0.0337		H6-N9 ^d^		0.0097	0.0290
V_FH_		C1-O6		0.0131		0.0478	V_GH_	O4-H7 ^a^		0.0194	0.0687
		O4-H8 ^b^		0.0136		0.0513		H5-O8 ^c^		0.0107	0.0404
							VI_GH_	O4-H10 ^b^		0.0143	0.0494
								H5-O8 ^c^		0.0126	0.0414
							VII_GH_	H6-O8 ^c^		0.0108	0.0370
								O4-H10 ^b^		0.0144	0.0499
							VIII_GH_	O4-H7 ^a^		0.0186	0.0690

^a^ OH···O path, ^b^ NH···O path, ^c^ CH···O path, ^d^ CH···N path.

**Table 5:** The comparison of wavenumber shifts (cm^−1^) of the vibrational bands of the hydroxylamine moiety in the complexes with formaldehyde, glyoxal, methylglyoxal, hydroxylamine, carbon monoxide and ammonia observed in argon and nitrogen matrices.

Approximate Description	HCHO		CHOCHO		CH_3_COCHO		NH_2_OH ^1^		CO ^2^	H_2_O ^3^		NH_3_^4^	
	Ar (p) ^5^	N_2_ (p) ^5^	Ar (n) ^5^	N_2_ (p,n) ^5^	Ar (n) ^5^	N_2_ (n) ^5^	Ar	N_2_	Ar	Ar	N_2_	Ar	N_2_
**ν(OH)**	−115	−125	−119	−97 (p) −119 (n)	−126	−137	−294 −345	−289 −333	−39	−93	−139	−297	−345
**δ(NOH)**	+70	+60	+61	+49 (p) +34 (n)	+53	+34	+119	+105		+10	+54	+121	+108
**ω(NH_2_)** **ωNH_2_**	+20	+24	+9	+10 (p)	+14	+7	+34	+31	+4	+19	+18	+12	+13
**ν(NO)**		+4	+5	+4 (p)	+8		+14	+9		+12	+19	+10	+8
**τ(OH)**	+162	+173					+362 +32	+340 +28				+422	+350

^1^ Data taken from ref [48]. ^2^ Data taken from ref [51]. ^3^ Data taken from ref [49]. ^4^ Data taken from ref [50]. ^5^ The wavenumbers shifts of the bands identified for the cyclic, planar (p) and nonplanar complexes (n).

## Data Availability

The data presented in this study are available in this article.

## Supplementary Materials

The following are available online, Figure S1: The optimized structures of the HCHO–NH_2_OH complexes; Figure S2: The optimized structures of the CHOCHO–NH_2_OH complexes; Figure S3: The spectra of the CHOCHO/Ar (a), ND_2_OD/Ar (b) and CHOCHO/ND_2_OD/Ar (c) matrices recorded after matrix deposition at 11 K; Figure S4: The spectra of the CHOCHO/N_2_ (a), ND_2_OD/N_2_ (b) and CHOCHO/ND_2_OD/N_2_ (c) matrices recorded after matrix deposition at 11 K; Figure S5: The optimized structures of the CH_3_COCHO–NH_2_OH complexes; Figure S6: The spectra of the CH_3_COCHO/Ar (a), ND_2_OD/Ar (b) and CH_3_COCHO/ND_2_OD/Ar (c) matrices recorded after matrix deposition at 11 K; Figure S7. The location of the bond (3,-1) and ring (3,1) critical points in the MP2/6-311++G(2d,2p) optimized structures of the HCHO–NH_2_OH complexes; Figure S8. The location of the bond (3,-1) and ring (3,1) critical points in the MP2/6-311++G(2d,2p) optimized structures of the CHOCHO–NH_2_OH complexes; Figure S9: The MP2 optimized structures of the formaldehyde, glyoxal and methylglyoxal complexes with hydroxylamine assigned to the structures isolated in argon matrix; Figure S10: The MP2 optimized structures of the formaldehyde, glyoxal and methylglyoxal complexes with hydroxylamine assigned to the structures isolated in nitrogen matrix; Table S1: Selected geometrical parameters of the hydroxylamine and formaldehyde subunits in their binary complexes; Table S2. Selected geometrical parameters of the hydroxylamine and glyoxal subunits in their binary complexes; Table S3: The comparison of the observed wavenumbers (cm^−1^) and wavenumber shifts (Δν = νGH − νM) for the CHOCHO–NH_2_OH (GH) complexes present in the Ar and N_2_ matrices with the corresponding calculated values for the complexes I_GH_–IV_GH_; Table S4: The comparison of the observed wavenumbers (cm^−1^) and wavenumber shifts (Δν = νGH − νM) for the CHOCHO–ND_2_OD (GH) complexes present in the Ar and N_2_ matrices with the corresponding calculated values for the complexes I_GH_–IV_GH_; Table S5: Selected geometrical parameters of the hydroxylamine and methylglyoxal subunits in their binary complexes; Table S6: The comparison of the observed wavenumbers (cm^−1^) and wavenumber shifts (Δν = νMH–νM) for the CH_3_COCHO–NH_2_OH (MH) complexes present in the Ar and N_2_ matrices with the corresponding calculated values for the complexes I_MHk_–IV_MHk_ and I_MHa_–IV_MHa_; Table S7: The comparison of the observed wavenumbers (cm^−1^) and wavenumber shifts (Δν = νMH – νM) for the CH_3_COCHO–ND_2_OD (MH) complexes present in the Ar and N_2_ matrices with the corresponding calculated values for the complexes I_MHk_–IV_MHk_ and I_MHa_–IV_MHa_.

## Sample Availability

Samples of the compounds are not available from the authors.
