# Supplementary material for: Complexes of Formaldehyde and α-Dicarbonyls with Hydroxylamine: FTIR Matrix Isolation and Theoretical Study
Source: Molecules. 2021 Feb 20;26(4):1144. doi: 10.3390/molecules26041144 (PMC7924657; doi:10.3390/molecules26041144)
Supplement: Supplementary file 1 [file molecules-26-01144-s001.pdf]

Supplementary Material

Complexes of formaldehyde and α-dicarbonyls with hydroxylamine: FTIR matrix isolation and theoretical study

Barbara Golec,[1*] Magdalena Sałdyka[2] and Zofia Mielke[2]

[1] *Institute of Physical Chemistry, Polish Academy of Sciences, Kasprzaka 44/52, 01-224 Warsaw, Poland*
[2] *Faculty of Chemistry, University of Wrocław, F. Joliot-Curie 14, 50-383 Wrocław, Poland*

Corresponding author:
dr Barbara Golec, e-mail: bgolec@ichf.edu.pl, Tel: + 48-22-343-3410.

This file contains:

Figure S1. The optimized structures of the HCHO-NH_2OH complexes. The ΔE^{CP}(ZPE) binding energies in kJ mol^{-1} are given in parentheses. The intermolecular distances are given in Å.

Figure S2. The optimized structures of the CHOCHO-NH_2OH complexes. The ΔE^{CP}(ZPE) binding energies in kJ mol^{-1} are given in parentheses. The intermolecular distances are given in Å.

Figure S3. The spectra of the CHOCHO/Ar (a), ND_2OD/Ar (b) and CHOCHO/ND_2OD/Ar (c) matrices recorded after matrix deposition at 11 K. The bands of CHOCHO-ND_2OD complexes are indicated by the arrows.

Figure S4. The spectra of the CHOCHO/N_2 (a), ND_2OD/N_2 (b) and CHOCHO/ND_2OD/N_2 (c) matrices recorded after matrix deposition at 11 K. The bands of CHOCHO-ND_2OD complexes are indicated by the arrows.

Figure S5. The optimized structures of the CH_3COCHO-NH_2OH complexes. The ΔE^{CP}(ZPE) binding energies in kJ mol^{-1} are given in parentheses. The intermolecular distances are given in Å.

Figure S6. The spectra of the CH_3COCHO/Ar (a), ND_2OD/Ar (b) and CH_3COCHO/ND_2OD/Ar (c) matrices recorded after matrix deposition at 11 K. The bands of CH_3COCHO-ND_2OD complexes are indicated by the arrows.

Figure S7. The location of the bond (3,-1) and ring (3,1) critical points in the MP2/6-311++G(2d,2p) optimized structures of the HCHO-NH_2OH complexes.

Figure S8. The location of the bond (3,-1) and ring (3,1) critical points in the MP2/6-311++G(2d,2p) optimized structures of the CHOCHO -NH_2OH complexes.

Figure S9. The MP2 optimized structures of the formaldehyde, glyoxal and methylglyoxal complexes with hydroxylamine assigned to the structures isolated in argon matrix. The intermolecular distances are given in Å. The binding energies in kJ mol^{-1} are given in parentheses.

Figure S10. The MP2 optimized structures of the formaldehyde, glyoxal and methylglyoxal complexes with hydroxylamine assigned to the structures isolated in nitrogen matrix. The intermolecular distances are given in Å. The binding energies in kJ mol^{-1} are given in parentheses.

Table S1. Selected geometrical parameters of the hydroxylamine and formaldehyde subunits in their binary complexes. For comparison the corresponding parameters of the monomers (M) are also given. The complexes are numbered in the same way as presented in Fig. S1. Bond distances are given in Å, angles in º.

Table S2. Selected geometrical parameters of the hydroxylamine and glyoxal subunits in their binary complexes. For comparison the corresponding parameters of the monomers (M) are also given. The complexes are numbered in the same way as presented in Fig. S2. Bond distances are given in Å, angles in º.

Table S3. The comparison of the observed wavenumbers (cm^{-1}) and wavenumber shifts (Δν = νGH – νM) for the CHOCHO-NH_2OH (GH) complexes present in the Ar and N_2 matrices with the corresponding calculated values for the complexes $\mathbf{I_{GH}}$ **-** $\mathbf{IV}_{GH}$.

Table S4. The comparison of the observed wavenumbers (cm^{-1}) and wavenumber shifts (Δν = νGH – νM) for the CHOCHO-ND_2OD (GH) complexes present in the Ar and N_2 matrices with the corresponding calculated values for the complexes $\mathbf{I_{GH}}$ **-** $\mathbf{IV}_{GH}$.

Table S5. Selected geometrical parameters of the hydroxylamine and methylglyoxal subunits in their binary complexes. For comparison the corresponding parameters of the monomers (M) are also given. The complexes are numbered in the same way as presented in Fig. S3. Bond distances are given in Å, angles in º.

Table S6. The comparison of the observed wavenumbers (cm^{-1}) and wavenumber shifts (Δν = νMH – νM) for the CH_3COCHO-NH_2OH (MH) complexes present in the Ar and N_2 matrices with the corresponding calculated values for the complexes $\mathbf{I_{MHk}}$ – $\mathbf{IV_{MHk}}$ and $\mathbf{I_{MHa}}$ **–** $\mathbf{IV_{MHa}}$.

Table S7. The comparison of the observed wavenumbers (cm^{-1}) and wavenumber shifts (Δν = νMH – νM) for the CH_3COCHO-ND_2OD (MH) complexes present in the Ar and N_2 matrices with the corresponding calculated values for the complexes $\mathbf{I_{MHk}}$ – $\mathbf{IV_{MHk}}$ and $\mathbf{I_{MHa}}$ **–** $\mathbf{IV_{MHa}}$.

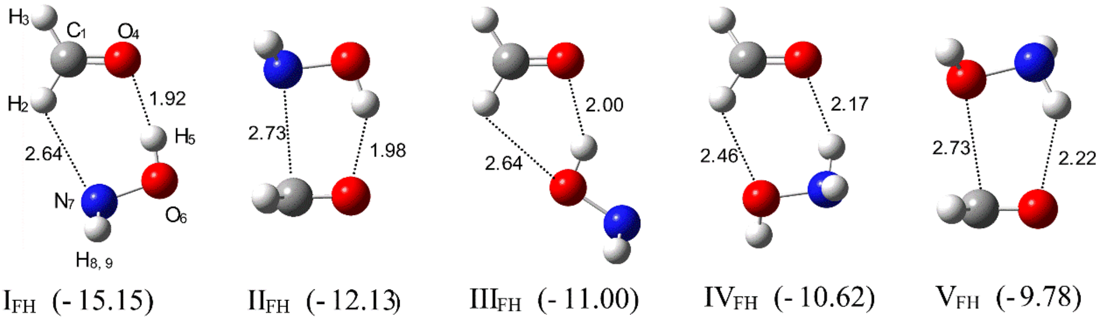

Figure S1. The optimized structures of the HCHO-NH_2OH complexes. The ΔE^{CP}(ZPE) binding energies in kJ mol^{-1} are given in parentheses. The intermolecular distances are given in Å.

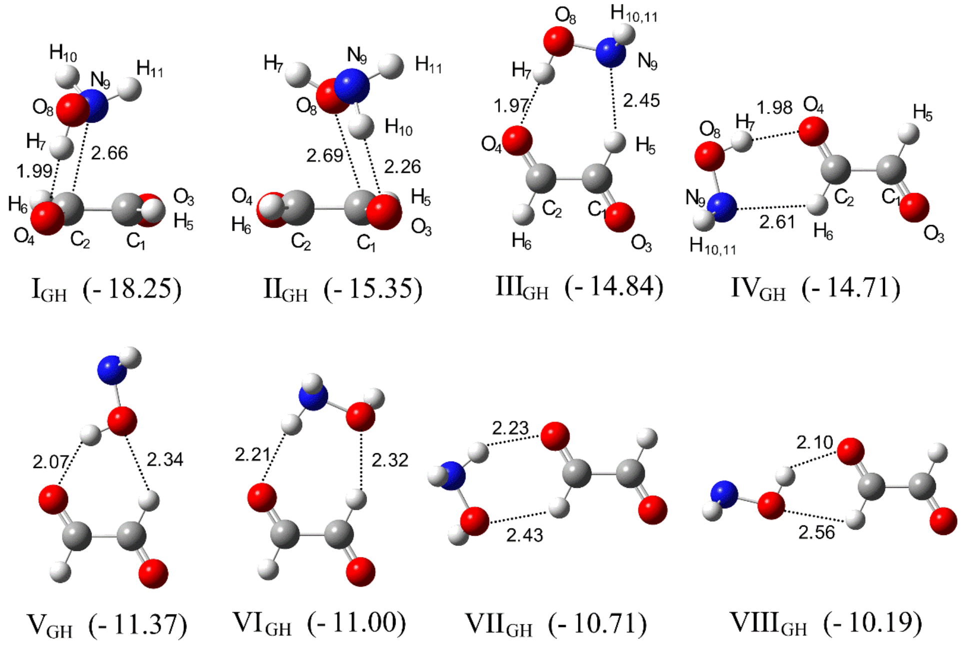

Figure S2. The optimized structures of the CHOCHO-NH_2OH complexes. The ΔE^{CP}(ZPE) binding energies in kJ mol^{-1} are given in parentheses. The intermolecular distances are given in Å.

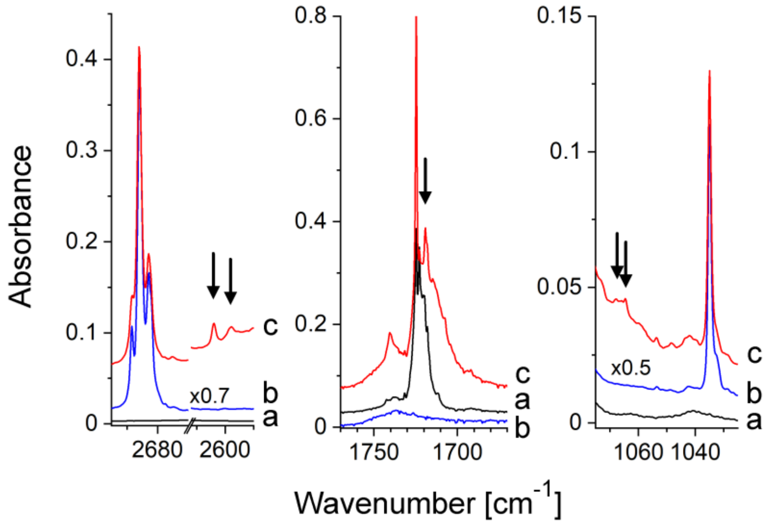

Figure S3. The spectra of the CHOCHO/Ar (a), ND_2OD/Ar (b) and CHOCHO/ND_2OD/Ar (c) matrices recorded after matrix deposition at 11 K. The bands of CHOCHO-ND_2OD complexes are indicated by the arrows..

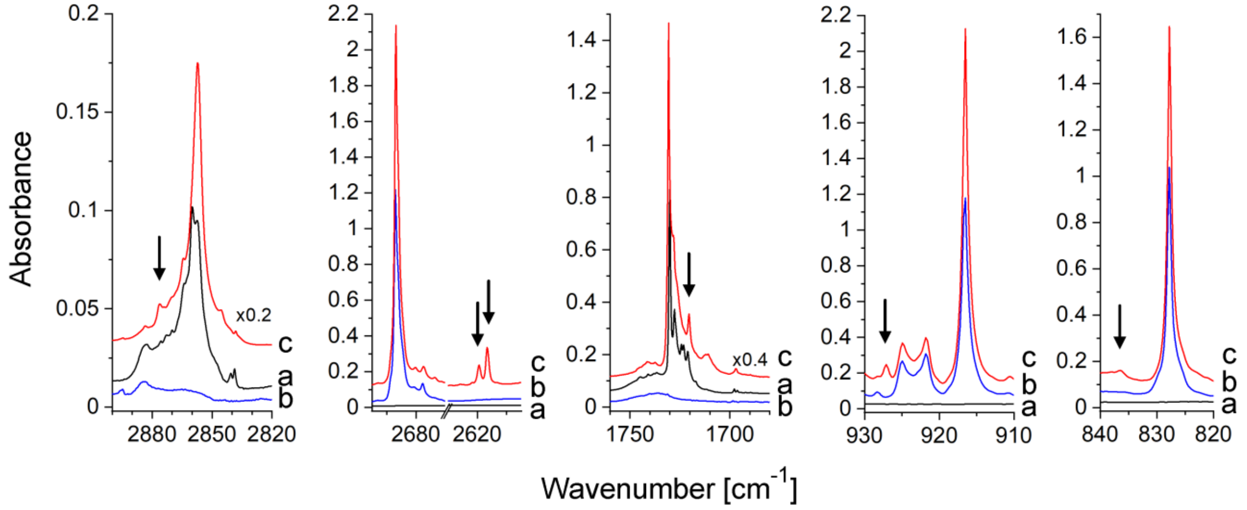

Figure S4. The spectra of the CHOCHO/N_2 (a), ND_2OD/N_2 (b) and CHOCHO/ND_2OD/N_2 (c) matrices recorded after matrix deposition at 11 K. The bands of CHOCHO-ND_2OD complexes are indicated by the arrows..

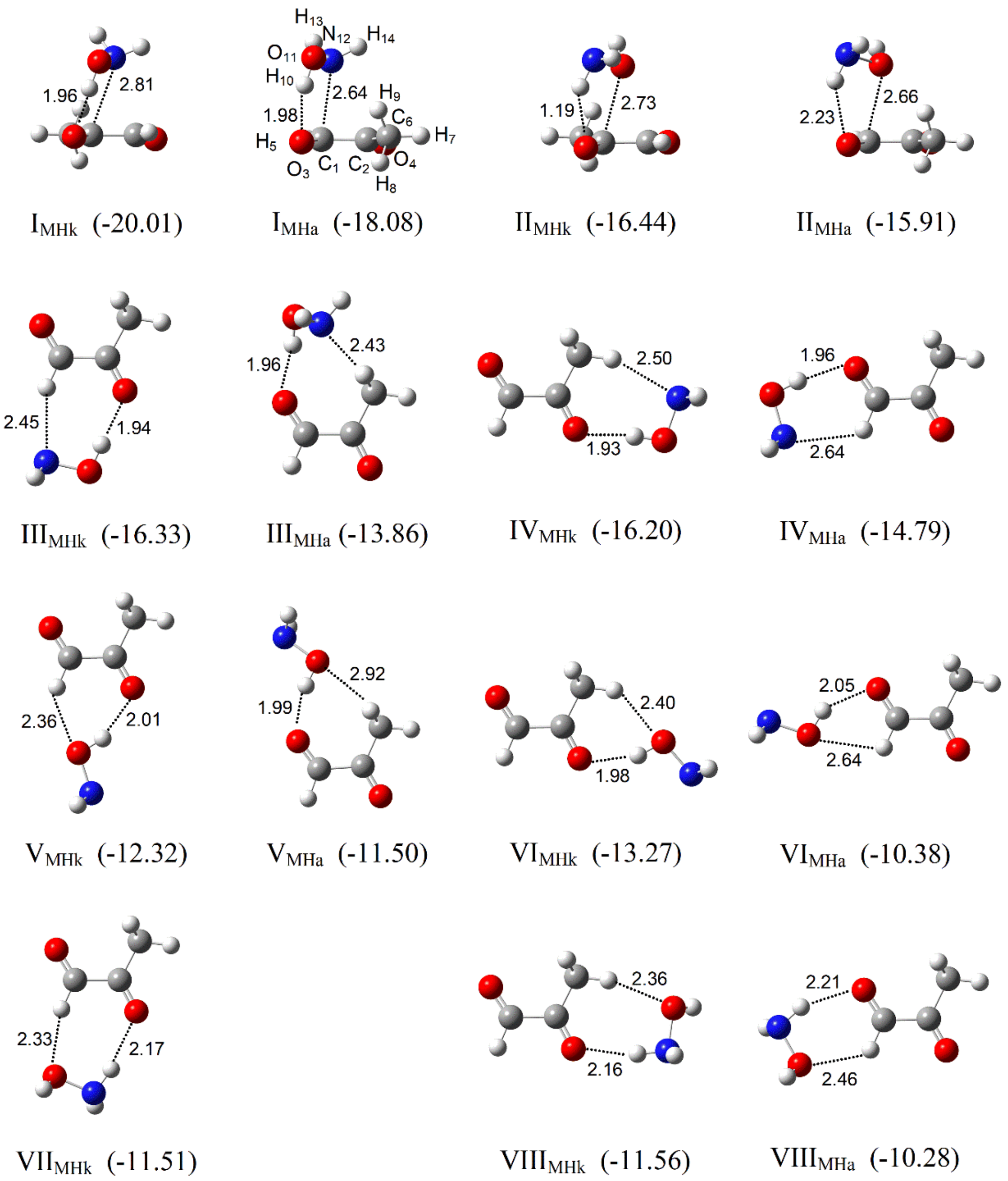

Figure S5. The optimized structures of the CH_3COCHO-NH_2OH complexes. The ΔE^{CP}(ZPE) binding energies in kJ mol^{-1} are given in parentheses. The intermolecular distances are given in Å.

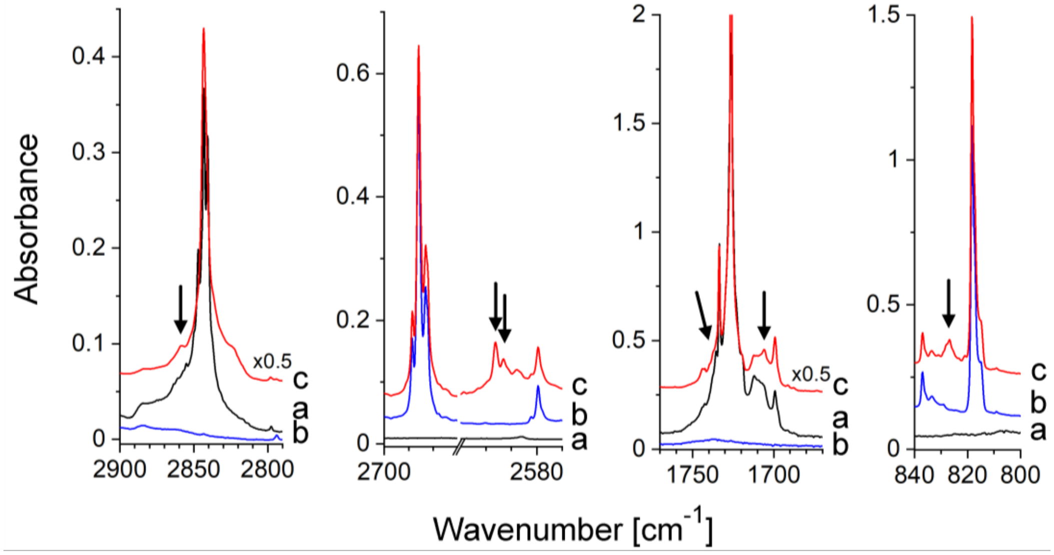

Figure S6. The spectra of the CH_3COCHO/Ar (a), ND_2OD/Ar (b) and CH_3COCHO/ND_2OD/Ar (c) matrices recorded after matrix deposition at 11 K. The bands of CH_3COCHO-ND_2OD complexes are indicated by the arrows.

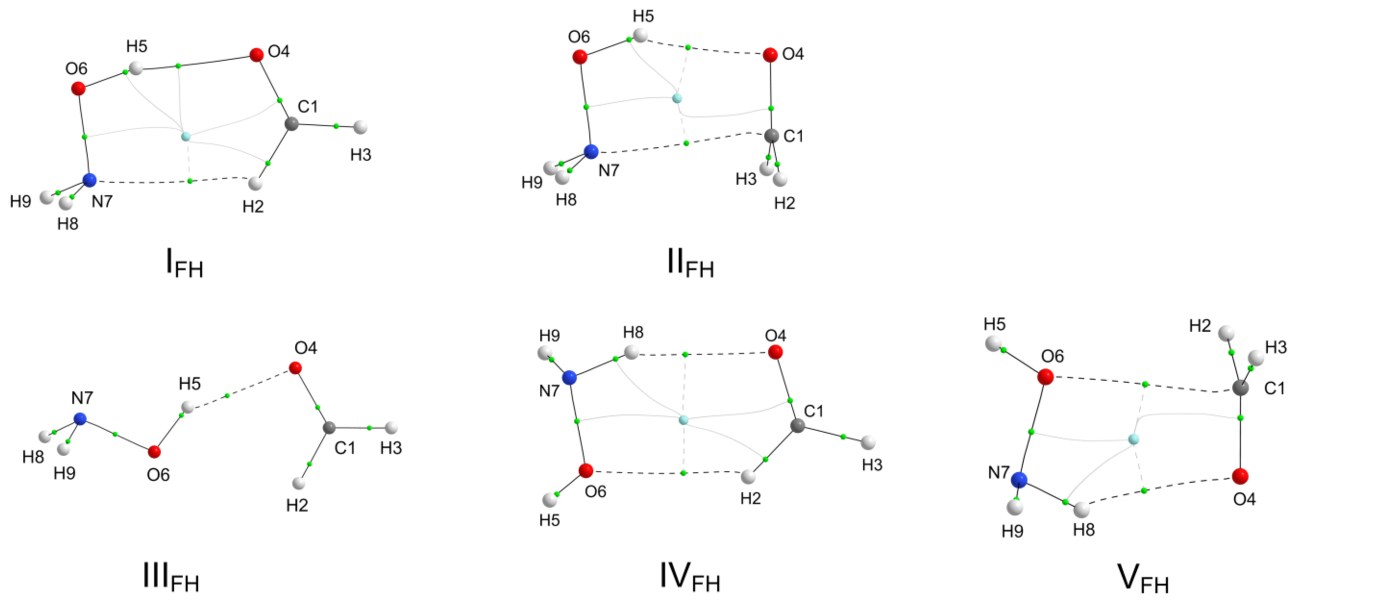

Figure S7. The location of the bond (3,-1) and ring (3,1) critical points in the MP2/6-311++G(2d,2p) optimized structures of the HCHO-NH_2OH complexes.

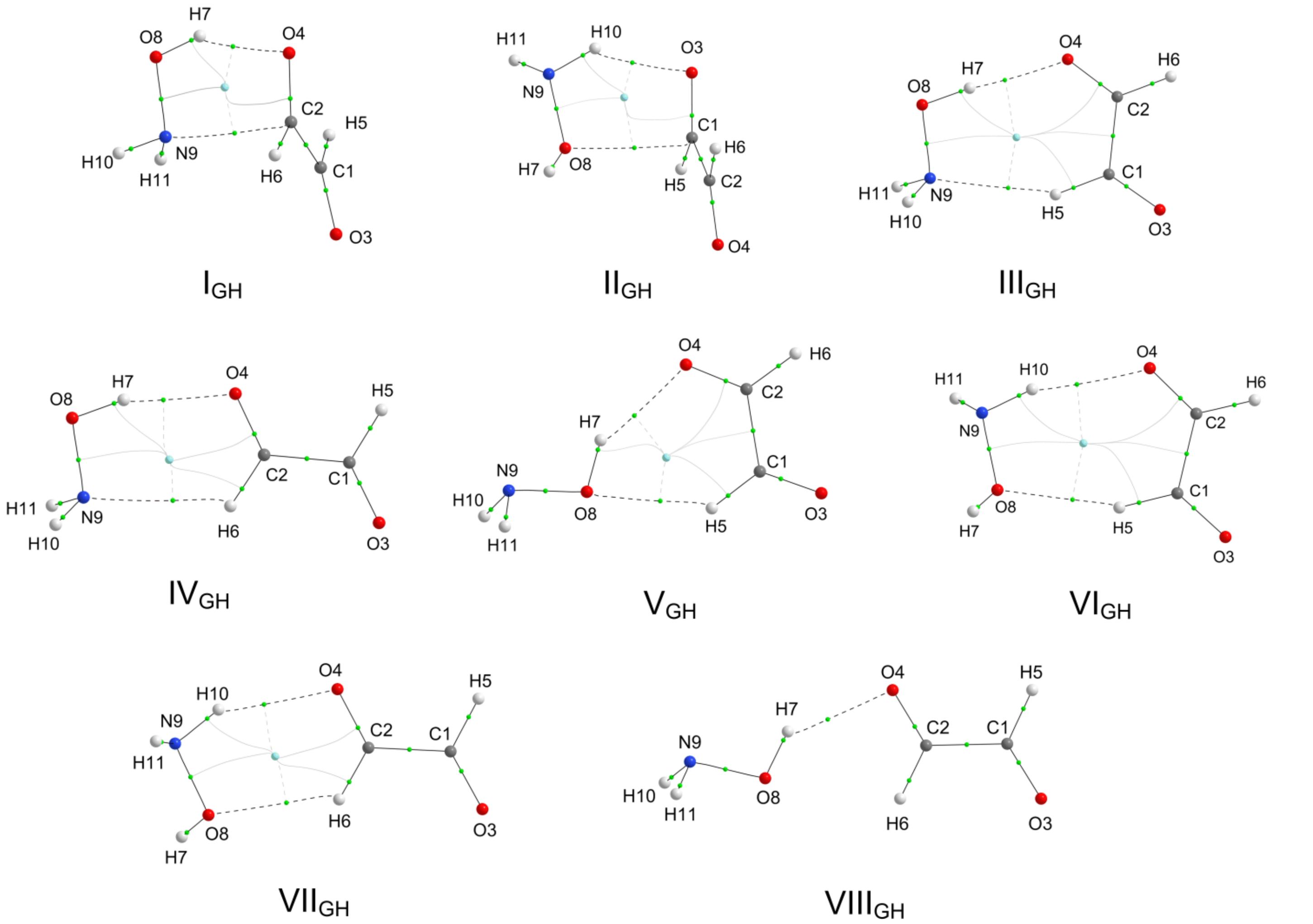

Figure S8. The location of the bond (3,-1) and ring (3,1) critical points in the MP2/6-311++G(2d,2p) optimized structures of the CHOCHO -NH_2OH complexes.

Argon matrix		
FA-HA	Gly-HA	MGly-HA
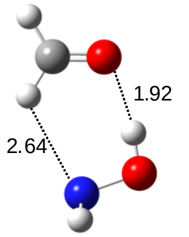	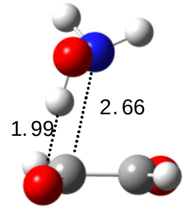	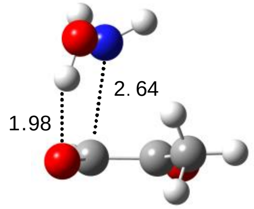
I_{FH} (-15.15)	I_{GH} (-18.25)	I_{MHa} (-18.08)

Figure S9. The MP2 optimized structures of the formaldehyde, glyoxal and methylglyoxal complexes with hydroxylamine assigned to the structures isolated in argon matrix. The intermolecular distances are given in Å. The binding energies in kJ mol^{-1} are given in parentheses.

Nitrogen matrix			
FA-HA	Gly-HA		MGly-HA
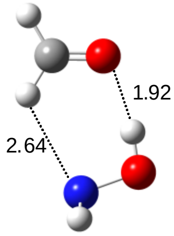	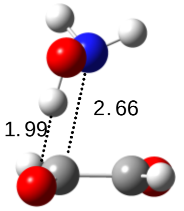	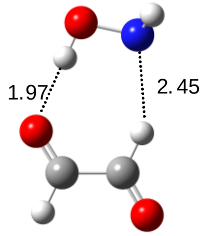	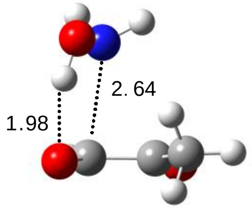
I_{FH} (-15.15)	I_{GH} (-18.25)	III_{GH} (-14.84)	I_{MHa} (-18.08)

Figure S10. The MP2 optimized structures of the formaldehyde, glyoxal and methylglyoxal complexes with hydroxylamine assigned to the structures isolated in nitrogen matrix. The intermolecular distances are given in Å. The binding energies in kJ mol^{-1} are given in parentheses.

Table S1. Selected geometrical parameters of the hydroxylamine and formaldehyde subunits in their binary complexes. For comparison the corresponding parameters of the monomers (M) are also given. The complexes are numbered in the same way as presented in Fig. S1. Bond distances are given in Å, angles in °.

Parameter	M	I_{FH}	II_{FH}	III_{FH}	IV_{FH}	V_{FH}
r C_1-O_4	1.213	1.219	1.218	1.217	1.218	1.217
r C_1-H_2	1.098	1.094	1.096	1.096	1.095	1.096
r C_1-H_3	1.098	1.097	1.096	1.096	1.098	1.097
r O_6-H_5	0.959	0.967	0.967	0.965	0.959	0.960
r O_6-N_7	1.448	1.447	1.439	1.446	1.455	1.451
r N_7-H_8	1.012	1.013	1.013	1.012	1.015	1.015
r N_7-H_9	1.012	1.013	1.013	1.012	1.012	1.012
R $O_4 \cdots H_5$		1.928	1.985	1.997		
R $H_2 \cdots N_7$		2.640				
R $H_2 \cdots O_6$				2.637	2.461	
R $H_8 \cdots O_4$					2.169	2.218
θ H_2-C_1-O_4	121.6	120.9	121.6	121.3	121.1	121.7
θ H_3-C_1-O_4	121.6	120.6	121.6	121.1	120.9	121.6
θ H_2-C_1-H_3	116.7	118.4	116.7	117.6	118.0	116.7
θ H_5-O_6-N_7	101.7	101.5	102.4	102.5	101.6	101.6
θ O_6-N_7-H_8	103.6	103.8	104.1	103.8	103.0	103.4
θ O_6-N_7-H_9	103.6	103.8	104.1	103.8	103.5	103.4
θ H_8-N_7-H_9	105.8	105.2	105.9	105.5	105.9	105.5
θ O_4-H_5-O_6		166.9	151.7	146.9		
θ C_1-H_2-N_7		120.0				
θ C_1-H_2-O_6					128.7	
θ O_4-H_8-N_7					156.7	141.3
φ H_5-O_6-N_7-H_8	124.8	125.1	124.6	124.9	128.2	125.5
φ H_5-O_6-N_7-H_9	-124.8	-125.1	-124.6	-124.9	-121.6	-124.6
φ C_1-O_4-H_5-O_6		-0.0	0.4	0.1		
φ C_1-H_2-N_7-O_6		-0.0				
φ C_1-O_4-H_8-N_7					0.0	-13.9
φ C_1-H_2-O_6-N_7					9.8	

Table S2. Selected geometrical parameters of the hydroxylamine and glyoxal subunits in their binary complexes. For comparison the corresponding parameters of the monomers (M) are also given. The complexes are numbered in the same way as presented in Fig. S2. Bond distances are given in Å, angles in º.

Parameter	M	I_{GH}	II_{GH}	III_{GH}	IV_{GH}	V_{GH}	VI_{GH}	VII_{GH}	VIII_{GH}
r C_1-C_2	1.518	1.514	1.515	1.519	1.517	1.520	1.519	1.517	1.517
r C_1-O_3	1.215	1.215	1.217	1.218	1.215	1.217	1.217	1.215	1.215
r C_2-O_4	1.215	1.220	1.215	1.219	1.220	1.217	1.217	1.219	1.219
r C_1-H_5	1.100	1.100	1.098	1.098	1.100	1.097	1.098	1.100	1.100
r C_2-H_6	1.100	1.099	1.100	1.099	1.096	1.099	1.100	1.097	1.098
r O_8-H_7	0.959	0.968	0.961	0.965	0.965	0.964	0.959	0.959	0.963
r O_8-N_9	1.448	1.436	1.451	1.446	1.447	1.447	1.453	1.454	1.447
r N_9-H_{10}	1.012	1.013	1.015	1.013	1.013	1.013	1.014	1.014	1.012
r N_9-H_{11}	1.012	1.013	1.013	1.013	1.013	1.013	1.013	1.013	1.012
R $O_4\cdots H_7$		1.988		1.970	1.980	2.070	2.209		2.096
R $O_3\cdots H_{10}$			2.264						
R $H_5\cdots N_9$				2.458	2.609				
R $O_8\cdots H_5$						2.337	2.321		
R $O_4\cdots H_{10}$								2.227	
R $O_8\cdots H_6$								2.428	2.559
θ C_2-C_1-O_3	121.3	121.7	121.3	119.9	121.3	119.7	119.9	121.5	121.3
θ C_1-C_2-O_4	121.3	121.6	121.7	122.0	120.3	122.4	122.0	120.4	120.7
θ C_2-C_1-H_5	115.4	115.0	115.5	115.9	115.4	116.5	116.1	115.3	115.3
θ C_1-C_2-H_6	115.4	115.3	115.0	115.6	117.3	115.0	115.3	117.0	116.4
θ O_3-C_1-H_5	123.3	123.3	123.1	124.3	123.3	123.7	124.0	123.2	123.3
θ O_4-C_2-H_6	123.3	123.0	123.3	122.4	122.4	122.6	122.7	122.6	122.8
θ H_7-O_8-N_9	101.7	102.5	102.0	102.2	101.5	102.9	129.1	101.7	102.6
θ O_8-N_9-H_{10}	103.6	104.8	103.2	103.8	103.8	103.8	103.2	102.8	103.8
θ O_8-N_9-H_{11}	103.6	104.4	103.4	103.8	103.8	103.8	103.2	103.5	103.8
θ H_{10}-N_9-H_{11}	105.8	106.2	105.3	105.4	105.3	105.6	105.9	105.9	105.6
θ C_2-O_4-H_7		100.8	100.7	126.6	112.0	117.1	125.8	100.3	
θ C_1-H_5-N_9			112.1	151.5					
θ C_2-O_4-N_9		93.6							
θ O_4-H_7-O_8		148.2		176.9	163.3	144.20	164.9	136.6	
θ O_3-H_9-N_9			135.5						
θ H_5-N_9-O_8				104.9					
θ H_5-N_9-H_{10}				118.5					
θ H_5-N_9-H_{11}				118.5					
θ C_2-H_6-N_9					118.9				
θ C_1-H_5-O_8						135.2	156.3		
θ O_4-H_{10}-N_9								151.6	
θ C_2-H_6-O_8									106.7
φ O_3-C_1-C_2-O_4	-180.0	166.0	-171.5	-180.0	180.0	-180.0	179.6	-180.0	180.0
φ O_3-C_1-C_2-H_6	0.0	-10.4	7.0	0.0	0.0	0.0	-0.4	0.0	0.0
φ H_5-C_1-C_2-O_4	0.0	-11.7	7.1	0.0	0.0	0.0	-0.6	0.0	0.0
φ H_7-O_8-N_9-H_{10}	124.8		130.1	125.0	125.1	124.9	128.4	-128.7	-124.9
φ H_7-O_8-N_9-H_{11}	-124.8		-120.3	-125.0	-125.1	-124.9	-121.3	121.0	124.8
φ C_2-O_4-H_7-O_8		10.9		0.0	-0.1	0.4	-1.1		-0.3
φ C_1-O_3-H_{10}-N_9			-26.0						
φ C_1-O_3-N_9-O_8			-3.5						
φ C_1-H_5-N_9-O_8				-0.1					
φ C_2-H_6-N_9-O_8					-0.1				
φ C_2-O_4-O_8-N_9		2.6							
φ C_1-H_5-O_8-H_7						-0.1			
φ C_1-H_5-O_8-N_9							18.3		
φ C_2-H_6-O_8-N_9								-8.1	-12.6
φ C_2-O_4-H_{10}-N_9								-1.7	

Table S3. The comparison of the observed wavenumbers (cm^{-1}) and wavenumber shifts (Δν = νGH – νM) for the CHOCHO-NH_2OH (GH) complexes present in the Ar and N_2 matrices with the corresponding calculated values for the complexes $\mathbf{I_{GH}}$ **-** $\mathbf{IV}_{GH}$.

**Approximate description**	**Experimental**						**Calculated**			
	**Ar**			**N_2**			**Δν**			
	**ν M**	**ν GH**	**Δν[1]**	**ν M**	**ν GH[2]**	**Δν[1]**	**I_{GH}**	**II_{GH}**	**III_{GH}**	**IV_{GH}**
NH_2OH										
**ν(OH)**	3635.5	3521.0<br>3512.4	-118.8	3637.6	3541.1<br>*3520.9*<br>*3515.8*	-96.5<br>*-119.2*	-145(77)	-19(66)	-106(383)	-103(356)
**δ(NOH)**	1351.2	1412.1<br>1410.2	+60.9	1367.4	1416.6<br>*1399.7*	+49.2<br>*+32.3*	+61(63)	-12(13)	+72(29)	+71(34)
**ω(NH_2)**	1118.3	1129.0<br>1125.8	+9.1	1133.0	1142.6	+9.6	+14(111)	+19(147)	+24(112)	+22(122)
**ν(NO)**	895.6			895.3	898.8	+3.5	+14(3)	-4(10)	+9(8)	+6(8)
CHOCHO										
**ν(CH)**	2860.1<br>2854.9	2857.6	-0.4	2857.1	2875.6	+18.5	-9(60)<br>+12(39)	-6(64)<br>+12(34)	+15(53)<br>+20(1)	+1(52)<br>+51(8)
**ν(C=O)**	1724.5	1719.0	-5.5	1730.1	1720.2<br>*1723.1*	-9.9<br>-7.0	-4(122)<br>+4(23)	+3(115)<br>+5(9)	-11(126)<br>-6(20)	-13(89)<br>-6(33)
**γ(CH)**	812.1<br>807.8			807.4	820.8	+13.4	+37(0)	+19(1)	+23(8)	+16(2)

[1] In the case when the splitting of the band was observed the average of the two wavenumbers at which the two peaks appear was taken into account to calculate Δν value.
[2] The wavenumbers in italic are due to complex of different structure (see text).

Table S4. The comparison of the observed wavenumbers (cm^{-1}) and wavenumber shifts (Δν = νGH – νM) for the CHOCHO-ND_2OD (GH) complexes present in the Ar and N_2 matrices with the corresponding calculated values for the complexes $\mathbf{I_{GH}}$ **-** $\mathbf{IV_{GH}}$.

**Approximate description**	**Experimental**						**Calculated**			
	**Ar**			**N_2**			**Δν**			
	**ν M**	**ν GH**	**Δν[1]**	**ν M**	**ν GH**	**Δν[1]**	**I_{GH}**	**II_{GH}**	**III_{GH}**	**IV_{GH}**
ND_2OD										
**ν(OD)**	2685.1	2604.0<br>2598.0	-84.1	2686.9	2619.2<br>2616.7	-68.9	-106(42)	-14(36)	-77(202)	-75(184)
**δ(NOD)**	1034.5	1068.0<br>1064.5	+33.5	1043.2			+38(6)	-7(3)	+38(7)	+41(7)
**ω(ND_2)**	915.0			916.5	927.1	+10.6	+0(29)	+12(44)	+14(40)	+14(42)
**ν(NO)**	818.4			827.6	838.0<br>836.6	+9.7	+28(41)	-10(52)	+21(40)	+16(44)
CHOCHO										
**ν(CH)**	2860.1<br>2854.9			2857.1	2876.3	+19.2	-10(59)<br>+12(36)	-6(64)<br>+12(34)	+15(48)<br>+20(1)	+1(51)<br>+51(9)
**ν(C=O)**	1724.5	1719.6	-4.9	1730.1	1720.5	-9.6	-5(125)<br>+4(25)	+3(127)<br>+5(9)	-11(128)<br>-6(20)	-13(93)<br>-6(33)
**γ(CH)**	812.1<br>807.8			807.4	820.9	+13.5	+41(5)	+19(2)	+22(4)	+16(3)

[1] In the case when the splitting of the band was observed the average of the two wavenumbers at which the two peaks appear was taken into account to calculate Δν value.

Table S5. Selected geometrical parameters of the hydroxylamine and methylglyoxal subunits in their binary complexes. For comparison the corresponding parameters of the monomers (M) are also given. The complexes are numbered in the same way as presented in Fig. S3. Bond distances are given in Å, angles in º.

Parameter	M	I_{MHk}	I_{MHa}	II_{MHk}	II_{MHa}	III_{MHk}	III_{MHa}	IV_{MHk}	IV_{MHa}
r C_1-C_2	1.528	1.524	1.524	1.524	1.524	1.528	1.528	1.527	1.526
r C_1-O_3	1.215	1.215	1.221	1.215	1.217	1.217	1.217	1.214	1.220
r C_2-O_4	1.221	1.225	1.221	1.223	1.221	1.225	1.222	1.225	1.097
r C_1-H_5	1.101	1.101	1.099	1.100	1.100	1.099	1.100	1.100	1.097
r C_2-C_6	1.499	1.495	1.499	1.497	1.497	1.496	1.494	1.492	1.498
r C_6-H_7	1.083	1.083	1.084	1.084	1.083	1.083	1.083	1.084	1.083
r C_6-H_8	1.088	1.088	1.088	1.088	1.087	1.088	1.090	1.089	1.088
r C_6-H_9	1.088	1.087	1.088	1.087	1.088	1.088	1.089	1.088	1.088
r O_{11}-H_{10}	0.959	0.968	0.968	0.960	0.961	0.966	0.965	0.967	0.965
r O_{11}-N_{12}	1.448	1.440	1.437	1.452	1.450	1.445	1.446	1.445	1.447
r N_{12}-H_{13}	1.012	1.013	1.013	1.016	1.016	1.013	1.013	1.013	1.013
r N_{12}-H_{14}	1.012	1.013	1.013	1.012	1.013	1.013	1.013	1.013	1.013
R O_4-H_{10}		1.956				1.936		1.930	
R O_3-H_{10}			1.980				1.961		1.964
R O_4-H_{13}				2.190					
R O_3-H_{13}					2.236				
R N_9-H_5						2.447			2.637
R N_9-H_8							2.432		
R N_9-H_7								2.501	
θ C_2-C_1-O_3	122.8	122.8	122.8	122.6	123.2	121.5	123.5	122.7	121.8
θ C_1-C_2-O_4	117.7	118.1	118.1	118.0	117.6	118.1	116.7	116.8	117.5
θ C_2-C_1-H_5	114.1	114.0	114.2	114.1	113.9	121.5	114.2	114.2	116.0
θ O_3-C_1-H_5	123.1	123.2	122.9	123.2	122.9	123.8	122.3	123.2	122.2
θ C_1-C_2-C_6	117.3	117.3	117.1	117.2	117.4	117.7	117.6	118.0	117.5
θ O_4-C_2-C_6	125.0	124.5	124.8	124.6	125.0	124.2	125.6	125.2	125.0
θ H_7-C_6-H_8	110.5	110.5	110.1	110.2	110.7	110.7	111.0	111.0	110.5
θ H_7-C_6-H_9	110.6	110.8	110.8	110.9	110.2	110.7	110.3	111.0	110.5
θ H_8-C_6-H_9	106.6	106.7	106.7	106.7	107.2	106.4	106.6	106.2	106.6
θ H_{10}-O_{11}-N_{12}	101.7	102.0	102.3	101.9	102.0	102.2	102.0	102.2	101.4
θ O_{11}-N_{12}-H_{13}	103.6	104.5	104.9	103.1	103.4	103.9	103.3	103.8	103.8
θ O_{11}-N_{12}-H_{14}	103.6	104.0	104.2	103.4	103.5	103.9	104.2	103.8	103.8
θ H_{13}-N_{12}-H_{14}	105.8	105.8	106.2	105.4	105.7	105.4	105.3	105.3	105.3
θ O_4-H_{10}-O_{11}		151.5				178.7		179.7	
θ O_3-H_{10}-O_{11}			148.6				171.7		164.5
θ C_1-O_3-H_{10}			99.9						112.2
θ O_4-H_{13}-N_{12}				139.7					
θ O_3-H_{13}-N_{12}					138.2				
θ C_1-H_5-N_9						155.2			118.5
θ C_6-H_8-N_9							153.38		
θ C_6-H_7-N_9								156.6	
φ O_3-C_1-C_2-O_4	180.0	169.3	169.1	170.2	172.3	-180.0	178.0	-180.0	-180.0
φ O_4-C_1-C_2-H_5	0.0	-8.1	-7.2	-8.0	-6.0	0.0	-1.9	0.0	0.0
φ H_5-C_1-C_2-C_6	-180.0	174.3	174.6	173.8	174.7	-180.0	178.5	-180.0	-180.0
φ O_3-C_1-C_2-C_6	0.0	-8.4	-9.1	-7.8	-7.0	0.0	-1.6	0.0	0.0
φ O_4-C_2-C_6-H_7	0.1	3.9	4.4	6.7	-3.1	0.0	1.4	0.3	0.0
φ O_4-C_2-C_6-H_8	-121.6	-117.6	-116.5	-114.3	-125.1	-121.8	-120.5	-121.8	-121.7
φ O_4-C_2-C_6-H_9	121.7	125.9	126.6	129.0	117.6	121.8	122.8	122.4	121.7
φ H_{10}-O_{11}-N_{12}-H_{13}	124.8	119.3	116.9	129.2	126.8	125.0	135.1	125.2	125.1
φ H_{10}-O_{11}-N_{12}-H_{14}	-124.8	-129.9	-131.7	-121.2	-123.1	-125.0	-115.1	-124.9	-125.0
φ O_4-H_{10}-O_{11}-N_{12}		-11.3						-54.3	
φ C_2-O_4-H_{10}-O_{11}		20.4				0.1		53.5	
φ O_3-H_{10}-O_{11}-N_{12}			3.4						-0.2
φ C_1-O_3-H_{10}-O_{11}			3.6				-96.8		0.2
φ O_4-H_{13}-N_{12}-O_{11}				8.9					
φ C_2-O_4-H_{13}-N_{12}				-26.1					
φ C_1-O_3-H_{13}-N_{12}					-26.2				-0.1
φ C_1-C_6-H_8-N_{12}							-95.8		
φ C_1-C_6-H_7-N_{12}								-0.4	

Table S5. - Continuation

Parameter	M	V_{MHk}	V_{MHa}	VI_{MHk}	VI_{MHa}	VII_{MHk}	VIII_{MHk}	VIII_{MHa}
r C_1-C_2	1.528	1.529	1.528	1.528	1.526	1.529	1.528	1.527
r C_1-O_3	1.215	1.216	1.217	1.214	1.219	1.218	1.215	1.219
r C_2-O_4	1.221	1.224	1.221	1.225	1.221	1.224	1.224	1.221
r C_1-H_5	1.101	1.098	1.100	1.100	1.099	1.099	1.101	1.097
r C_2-C_6	1.499	1.496	1.496	1.495	1.498	1.497	1.495	1.499
r C_6-H_7	1.083	1.083	1.083	1.084	1.083	1.083	1.084	1.083
r C_6-H_8	1.088	1.088	1.088	1.088	1.088	1.088	1.089	1.088
r C_6-H_9	1.088	1.088	1.088	1.088	1.088	1.088	1.088	1.088
r O_{11}-H_{10}	0.959	0.965	0.964	0.965	0.964	0.959	0.959	0.959
r O_{11}-N_{12}	1.448	1.446	1.447	1.446	1.447	1.454	1.454	1.454
r N_{12}-H_{13}	1.012	1.013	1.013	1.013	1.012	1.012	1.013	1.014
r N_{12}-H_{14}	1.012	1.013	1.013	1.013	1.012	1.015	1.015	1.013
R O_4-H_{10}		2.005		1.978				
R O_{11}-H_5		2.359			2.642	2.331		2.460
R O_3-H_{10}			1.990		2.047			
R O_{11}-H_9			2.539					
R O_{11}-H_7				2.401			2.362	
R O_4-H_{14}						2.166	2.157	
R O_3-H_{13}								2.207
θ C_2-C_1-O_3	122.8	121.6	123.6	122.7	122.2	121.5	122.8	122.0
θ C_1-C_2-O_4	117.7	118.4	116.7	116.9	117.5	118.1	117.0	117.7
θ C_2-C_1-H_5	114.1	115.1	114.0	114.1	115.1	114.9	114.1	115.6
θ O_3-C_1-H_5	123.1	123.3	122.4	123.2	122.6	123.6	123.1	122.4
θ C_1-C_2-C_6	117.3	117.4	118.0	117.7	117.3	117.5	117.7	117.4
θ O_4-C_2-C_6	125.0	124.2	125.3	125.4	125.1	124.4	125.3	124.9
θ H_7-C_6-H_8	110.5	110.6	110.4	110.7	110.5	110.6	110.8	110.5
θ H_7-C_6-H_9	110.6	110.7	110.5	110.7	110.5	110.7	110.9	110.5
θ H_8-C_6-H_9	106.6	106.5	106.1	106.3	106.6	106.4	106.2	106.5
θ H_{10}-O_{11}-N_{12}	101.7	102.9	102.3	102.7	102.6	101.7	101.6	101.7
θ O_{11}-N_{12}-H_{13}	103.6	103.8	103.8	103.8	103.8	103.5	103.5	102.9
θ O_{11}-N_{12}-H_{14}	103.6	103.8	103.8	103.8	103.8	103.2	103.2	103.5
θ H_{13}-N_{12}-H_{14}	105.8	105.5	105.5	105.5	105.5	105.9	105.9	106.0
θ O_4-H_{10}-O_{11}		151.0		155.0				
θ C_1-O_3-H_{10}		138.1			101.3			
θ O_3-H_{10}-O_{11}			162.9		142.4			
θ C_2-H_9-O_{11}			119.2					
θ C_2-H_7-O_{11}				139.6			163.2	
θ C_1-H_5-O_{11}					104.8			125.7
θ O_4-H_{14}-N_{12}						167.4	169.5	
θ O_3-H_{13}-N_{12}								153.2
φ O_3-C_1-C_2-O_4	180.0	180.0	-178.1	180.0	180.0	-180.0	179.8	179.8
φ O_4-C_1-C_2-H_5	0.0	0.0	1.8	0.0	0.0		-0.1	0.0
φ H_5-C_1-C_2-C_6	-180.0	180.0	-178.2	180.0	180.0	-180.0	-180.0	179.9
φ O_3-C_1-C_2-C_6	0.0	0.0	1.9	0.0	0.0	0.0	0.0	-0.1
φ O_4-C_2-C_6-H_7	0.1	0.0	-0.7	-0.1	0.0	0.2	2.0	0.0
φ O_4-C_2-C_6-H_8	-121.6	-121.8	-122.2	-122.1	-121.6	-121.6	-120.0	-121.6
φ O_4-C_2-C_6-H_9	121.7	121.8	121.2	121.9	121.6	122.0	124.2	121.6
φ H_{10}-O_{11}-N_{12}-H_{13}	124.8	124.9	124.9	124.9	124.9	121.4	121.8	128.5
φ H_{10}-O_{11}-N_{12}-H_{14}	-124.8	-124.9	-125.0	-124.9	-124.9	-128.4	-128.0	-121.3
φ O_4-H_{10}-O_{11}-N_{12}		179.9		-179.9				
φ C_2-O_4-H_{10}-O_{11}		0.0		0.0				
φ O_3-H_{10}-O_{11}-N_{12}			164.4					
φ C_1-O_3-H_{10}-O_{11}			2.9		-0.3			
φ C_2-C_6-H_7-O_{11}				0.3			-0.7	
φ C_1-H_5-O_{11}-H_{10}					-0.3			
φ C_2-O_4-H_{14}-N_{12}						1.5	3.0	
φ C_2-C_1-H_5-O_{11}						12.2		
φ C_1-O_3-H_{13}-N_{12}								2.7
φ O_3-C_1-H_5-O_{11}								-4.8

Table S6. The comparison of the observed wavenumbers (cm^{-1}) and wavenumber shifts (Δν = νMH – νM) for the CH_3COCHO-NH_2OH (MH) complexes present in the Ar and N_2 matrices with the corresponding calculated values for the complexes $\mathbf{I_{MHk}}$ – $\mathbf{IV_{MHk}}$ and $\mathbf{I_{MHa}}$ – $\mathbf{IV_{MHa}}$.

**Approximate description**	**Experimental**						**Calculated**							
	**Ar**			**N_2**			**Δν**							
	**ν M**	**ν MH**	**Δν[1]**	**ν M**	**ν MH**	**Δν[1]**	**I_{MHk}**	**II_{MHk}**	**III_{MHk}**	**IV_{MHk}**	**I_{MHa}**	**II_{MHa}**	**III_{MHa}**	**IV_{MHa}**
NH_2OH														
**ν(OH)**	3635.5	3511.7<br>3507.6	-125.8	3637.6	3505.7<br>3495.2	-137.1	-148(138)	-14(62)	-127(494)	-132(520)	-156(73)	-17(68)	-92(367)	-112(402)
**δ(NOH)[2]**	1351.2	1404.1	+52.9	1367.4	1402.7<br>1400.3	+34.1	+64(58)	-13(12)	+81(28)	+86(27)	+62(51)	-17(17)	+64(31)	+74(33)
**ω(NH_2)**	1118.3	1134.1<br>1131.2	+14.4	1133.0	1139.3<br>1140.6	+7.0	+17(117)	+19(148)	+23(109)	+21(105)	+11(109)	+13(152)	+22(110)	+21(120)
**ν(NO)**	895.6	904.8<br>902.9	+8.3	895.3			+9(5)	-4(7)	+8(8)	+6(7)	+13(6)	-2(7)	+7(7)	+5(8)
CH_3COCHO														
**ν(CH)**	2843.1<br>2840.7	2858.2	+16.3	2844.7<br>2840.2<br>2835.9			-1(60)	+8(60)	+20(1)	+4(60)	+24(51)	+5(57)	+17(57)	+57(11)
**ν_{ket}(C=O)**	1733.5	1737.7	+4.2	1739.0<br>1737.2<br>1735.9	1741.3	+3.9	-5(132)	0(134)	-3(64)	-2(18)	+4(61)	0(53)	-4(38)	-3(80)
**ν_{ald}(C=O)**	1726.4	1705.4	-21.0	1730.1<br>1727.9	1710.8	-18.2	+6(27)	+7(11)	-14(126)	-4(145)	-10(97)	+2(88)	0(137)	-17(65)
**δ(CH_3)[2]**	1420.0	1416.1	-3.9	1423.2<br>1420.8			0(8)<br>+3(24)<br>-3(36)	-1(8)<br>-1(18)<br>-3(32)	-2(10)<br>-2(20)<br>+2(36)	+3(8)<br>-2(10)<br>+7(32)	+5(6)<br>+3(25)<br>-2(31)	+4(8)<br>0(11)<br>-5(29)	+13(9)<br>+6(10)<br>+10(25)	+1(10)<br>0(15)<br>0(30)
**ν_{as}(C-C)**	1228.3	1233.7	+5.4	1234.5<br>1229.9	1240.5<br>1237.1	+6.6	+5(14)	+9(16)	+8(13)	+15(20)	+5(15)	+6(15)	+3(16)	+1(16)
**ν_{s}(C-C)**	777.1	784.7	+7.6	780.9<br>779.5<br>777.6	785.8<br>782.3	+4.8	+6(15)	+6(13)	+4(13)	+6(13)	+6(15)	+5(11)	+5(14)	+2(20)

[1] In the case when the splitting of the band was observed the average of the two wavenumbers at which the two peaks appear was taken into account to calculate Δν value.

2 The 1416.1 and 1404.1 cm^{-1} bands observed in the spectra of the complex in Ar matrix are assigned to the coupled δ(NOH)+δ(CH_3) vibrations.

Table S7. The comparison of the observed wavenumbers (cm^{-1}) and wavenumber shifts ($\Delta\nu = \nu MH - \nu M$)) for the CH_3COCHO-ND_2OD (MH) complexes present in the Ar and N_2 matrices with the corresponding calculated values for the complexes $\mathbf{I_{MHk}}$ – $\mathbf{IV_{MHk}}$ and $\mathbf{I_{MHa}}$ – $\mathbf{IV_{MHa}}$.

**Approximate description**	**Experimental**						**Calculated**							
	**Ar**			**N_2**			**Δν**							
	**ν M**	**ν MH**	**Δν[1]**	**ν M**	**ν MH**	**Δν[1]**	$\mathbf{I_{MHk}}$	$\mathbf{II_{MHk}}$	$\mathbf{III_{MHk}}$	$\mathbf{IV_{MHk}}$	$\mathbf{I_{MHa}}$	$\mathbf{II_{MHa}}$	$\mathbf{III_{MHa}}$	$\mathbf{IV_{MHa}}$
ND_2OD														
**ν(OD)**	2685.1	2596.4<br>2593.2	-90.3	2686.9	2595.6<br>2593.5	-92.3	-109(73)	-10(34)	-93(253)	-96(264)	-114(40)	-12(37)	-67(189)	-61(207)
**ν(NO)**	818.4	821.1	+2.7	827.6			+22(44)	-10(50)	+22(38)	+23(37)	+23(33)	-8(50)	+14(33)	+16(44)
CH_3COCHO														
**ν(CH)**	2843.1<br>2840.7	2858.6	+16.7				-2(58)	+8(59)	+20(1)	+4(56)	+24(51)	+6(57)	+17(54)	+57(12)
**ν_{ket}(C=O)**	1733.5	1737.0	+3.5	1739.0<br>1737.2<br>1735.9			-6(137)	0(144)	-3(63)	-7(144)	+3(59)	+3(52)	-4(37)	-3(79)
**ν_{ald}(C=O)**	1726.4	1705.9	-20.5	1730.1<br>1727.9			+6(30)	+7(12)	-14(128)	+1(19)	-10(103)	+1(102)	0(139)	-17(70)
**δ(CH_3)**	1420.0			1366.1<br>1364.5<br>1368.2	1369.2	+2.9	+6(1)	+4(1)	+27(3)	+27(3)	+4(7)<br>+3(14)<br>-2(33)	+4(8)<br>0(12)<br>-5(34)	0(16)<br>+6(21)<br>+10(23)	+1(10)<br>0(16)<br>0(30)
**ν_{as}(C-C)**	1228.3			1234.5<br>1229.9	1241.4<br>1238.8	+7.9	+13(18)	+9(16)	+8(14)	+16(22)	+6(17)	+6(16)	+3(15)	+1(16)
**ν_s(C-C)**	777.1			780.9<br>779.5<br>777.6	785.6<br>783.7	+5.4	+8(11)	+5(12)	+4(13)	+5(13)	+5(12)	+3(11)	+5(13)	+2(21)

[1] In the case when the splitting of the band was observed the average of the two wavenumbers at which the two peaks appear was taken into account to calculate Δν value.
